# Supplementary material for: Long Noncoding RNA SBF2-AS1 Is Critical for Tumorigenesis of Early-Stage Lung Adenocarcinoma
Source: Mol Ther Nucleic Acids. 2019 Apr 13;16:543–53. doi: 10.1016/j.omtn.2019.04.004 (PMC6506611; doi:10.1016/j.omtn.2019.04.004)
Supplement: Document S1. Figures S1–S4 and Data S1 [file mmc1.pdf]

OMTN, Volume *16*

Supplemental Information

Long Noncoding RNA SBF2-AS1 Is Critical for Tumorigenesis of Early-Stage Lung Adenocarcinoma

Rui Chen, Wenjia Xia, Siwei Wang, Youtao Xu, Zhifei Ma, Weizhang Xu, Erbao Zhang, Jie Wang, Tian Fang, Quan'an Zhang, Gaochao Dong, William Chi-shing Cho, Patrick C. Ma, Giovanni Brandi, Simona Tavolari, Peter Ujhazy, Giulio Metro, Helmut H. Popper, Rong Yin, Mantang Qiu, and Lin Xu

Supplementary Materials

Supplementary Table1. Primers and siRNA sequences

Supplementary Table2. Differentially expressed genes after knockdown of SBF2-AS1

Supplementary Table3. Function enrichment analyses of differentially expressed genes after knockdown of SBF2-AS1

Supplementary Table4. miRNA binding sites within SBF2-AS1 sequence predicted by miRanda algorithm and CLIP-seq

Supplementary Table5. Cell cycle-related genes that were downregulated after knockdown of SBF2-AS1

Supplementary Table6. Co-expression between 19 cell cycle-related genes and SBF2-AS1 among cancers

Supplementary File 1. Constructed plasmids vectors used in this study.

The following plasmids were generated by inserting the indicated sequences in the Pezx-FR02 dual- Luciferase reporter vector.

E2F1-WT, wild type E2F1 3'UTR
CAGGGCTTGGAGGGACCAGGGTTTCCAGAGATGCTCACCTTGTCTCTGCA
GCCCTGGAGCCCCCTGTCCCTGGCCGTCCTCCCAGCCTGTTTGGAAACATT
TAATTTATACCCCTCTCCTCTGTCTCCAGAAGCTTCTAGCTCTGGGGTCTGG
CTACCGCTAGGAGGCTGAGCAAGCCAGGAAGGGAAGGAGTCTGTGTGGTG
TGTATGTGCATGCAGCCTACACCCACACGTGTGTACCGGGGGTGAATGTGT
GTGAGCATGTGTGTGTGCATGTACCGGGGAATGAAGGTGAACATACACCTC
TGTGTGTGCACTGCAGACACGCCCCAGTGTGTCCACATGTGTGTGCATGAG
TCCATGTGTGCGCGTGGGGGGGCTCTAACTGCACTTTCGGCCCTTTTGCTC
TGGGGGTCCCACAAGGCCCAGGGCAGTGCCTGCTCCCAGAATCTGGTGCT
CTGACCAGGCCAGGTGGGGAGGCTTTGGCTGGCTGGGCGTGTAGGACGGT
GAGAGCACTTCTGTCTTAAAGGTTTTTTCTGATTGAAGCTTTAATGGAGCGT
TATTTATTTATCGAGGCCTCTTTGGTGAGCCTGGGGAATCAGCAAAGGGGA
GGAGGGGTGTGGGGTTGATACCCCAACTCCCTCTACCCTTGAGCAAGGGC
AGGGGTCCCTGAGCTGTTCTTCTGCCCCATACTGAAGGAACTGAGGCCTGG
GTGATTTATTTATTGGGAAAGTGAGGGAGGGAGACAGACTGACTGACAGC
CATGGGTGGTCAGATGGTGGGGTGGGCCCTCTCCAGGGGGCCAGTTCAGG
GCCCCAGCTGCCCCCCAGGATGGATATGAGATGGGAGAGGTGAGTGGGGG
ACCTTCACTGATGTGGGCAGGAGGGGTGGTGAAGGCCTCCCCCAGCCCAG
ACCCTGTGGTCCCTCCTGCAGTGTCTGAAGCGCCTGCCTCCCCACTGCTCT
GCCCCACCCTCCAATCTGCACTTTGATTTGCTTCCTAACAGCTCTGTTCCCT
CCTGCTTTGGTTTTAATAAATATTTTGATGACGTT

E2F1-MUT1, binding site of miR-338-3p was deletion-mutated
CAGGGCTTGGAGGGACCAGGGTTTCCAGAGATGCTCACCTTGTCTCTGCA
GCCCTGGAGCCCCCTGTCCCTGGCCGTCCTCCCAGCCTGTTTGGAAACATT
TAATTTATACCCCTCTCCTCTGTCTCCAGAAGCTTCTAGCTCTGGGGTCTGG
CTACCGCTAGGAGGCTGAGCAAGCCAGGAAGGGAAGGAGTCTGTGTGGTG
TGTATGTGCATGCAGCCTACACCCACACGTGTGTACCGGGGGTGAATGTGT
GTGAGCATGTGTGTGTGCATGTACCGGGGAATGAAGGTGAACATACACCTC
TGTGTGTGCACTGCAGACACGCCCCAGTGTGTCCACATGTGTGTGCATGAG
TCCATGTGTGCGCGTGGGGGGGCTCTAACTGCACTTTCGGCCCTTTTGCTC
TGGGGGTCCCACAAGGCCCAGGGCAGTGCCTGCTCCCAGAATCTGGTGCT
CTGACCAGGCCAGGTGGGGAGGCTTTGGCTGGCTGGGCGTGTAGGACGGT
GAGAGCACTTCTGTCTTAAAGGTTTTTTCTGATTGAAGCTTTAATGGAGCGT
TATTTATTTATCGAGGCCTCTTTGGTGAGCCTGGGGAATCAGCAAAGGGGA
GGAGGGGTGTGGGGTTGATACCCCAACTCCCTCTACCCTTGAGCAAGGGC

AGGGGTCCCTGAGCTGTTCTTCTGCCCCATACACTGAGGCCTGGGTGATTT
ATTTATTGGGAAAGTGAGGGAGGGAGACAGACTGACTGACAGCCATGGGT
GGTCAGATGGTGGGGTGGGCCCTCTCCAGGGGGCCAGTTCAGGGCCCCAG
CTGCCCCCCAGGATGGATATGAGATGGGAGAGGTGAGTGGGGGACCTTCA
CTGATGTGGGCAGGAGGGGTGGTGAAGGCCTCCCCCAGCCCAGACCCTGT
GGTCCCTCCTGCAGTGTCTGAAGCGCCTGCCTCCCCACTGCTCTGCCCCAC
CCTCCAATCTGCACTTTGATTTGCTTCCTAACAGCTCTGTTCCCTCCTGCTT
TGGTTTTAATAAATATTTTGATGACGTT

E2F1-MUT2, binding sites of miR-362-3p were deletion-mutated
CAGGGCTTGGAGGGACCAGGGTTTCCAGAGATGCTCACCTTGTCTCTGCA
GCCCTGGAGCCCCCTGTCCCTGGCCGTCCTCCCAGCCTGTTTGGAAACATT
TAATTTATACCCCTCTCCTCTGTCTCCAGAAGCTTCTAGCTCTGGGGTCTGG
CTACCGCTAGGAGGCTGAGCAAGCCAGGAAGGGAAGGAGTCTGTGTGTGT
GCATGCAGCCTACACCCACACCCGGGGGTGAATGTGTGTGAGCATGTGTGT
GTGCATGTACCGGGGAATGAAGGTGAACATACACCTCTGTGTGTGCACTGC
AGACACGCCCCAGTGTGTCCACATGTGTGTGCATGAGTCCATGTGTGCGCG
TGGGGGGGCTCTAACTGCACTTTCGGCCCTTTTGCTCTGGGGGTCCCACAA
GGCCCAGGGCAGTGCCTGCTCCCAGAATCTGGTGCTCTGACCAGGCCAGG
TGGGGAGGCTTTGGCTGGCTGGGCGTGTAGGACGGTGAGAGCACTTCTGT
CTTAAAGGTTTTTTCTGATTGAAGCTTTAATGGAGCGTTATTTATTTATCGAG
GCCTCTTTGGTGAGCCTGGGGAATCAGCAAAGGGGAGGAGGGGTGTGGG
GTTGATACCCCAACTCCCTCTACCCTTGAGCAAGGGCAGGGGTCCCTGAGC
TGTTCTTCTGCCCCATACTGAAGGAACTGAGGCCTGGGTGATTTATTTATTG
GGAAAGTGAGGGAGGGAGACAGACTGACTGACAGCCATGGGTGGTCAGA
TGGTGGGGTGGGCCCTCTCCAGGGGGCCAGTTCAGGGCCCCAGCTGCCCC
CCAGGATGGATATGAGATGGGAGAGGTGAGTGGGGGACCTTCACTGATGT
GGGCAGGAGGGGTGGTGAAGGCCTCCCCCAGCCCAGACCCTGTGGTCCCT
CCTGCAGTGTCTGAAGCGCCTGCCTCCCCACTGCTCTGCCCCACCCTCCAA
TCTGCACTTTGATTTGCTTCCTAACAGCTCTGTTCCCTCCTGCTTTGGTTTTA
ATAAATATTTTGATGACGTT

The following plasmids were generated by inserting the indicated sequences between the BamHI site and XhoI sites of pcDNA3.1(+) (Life Technologies).

SBF2-AS1 WT, wild SBF2-AS1 full length sequence
CAGGTTCCAGCCCCGACCCGGGCGCGCGGGGCCGACTAGGGTCGGGTCCA
GTGTGCGGTGGTCGCTCCGCTCCGGGCCGCTCCGCTCTGGGCGTCAGGGC
GCGGGGAGCTGCCCCGGGGTTCTGTCCACCGGGGAGGAAAGCCACGAGC
ACTGAGCGCCTCCTGAGAGCCAGCCCTGACGTGAACTCATTTTATCTGCCA

CGACCCAGAAGGAGTCTACTGCTAAGATTTCAGCATGTCCTGTGGCTGAG
TTAATCAGAGTTATGACAGGAAGGTACCGGGCACACCATCGCAATGCTCC
ATCAATGCTAGTATGTTGTGTTCTTTCCTTCATATCAAGTCAACTCAAGCT
TGCTCTACTTACCTGGTGTACACAGTCTAAGAACTGTAAGAAGACTGGAG
CAAAACCACTCCCCTGACAGTTGAGGGTCAAGCTGCTCCTCTGACTGAAT
TTGTGACCAAAAGAGAGCCACTCTTTTTCAACCAACATCTGGAAGCCTTC
AAGTGTCCTATAAAAGGGATCACTGAGTAACTGAACCAGGGATGTCACCT
AGGGCATAAGCAGGATGGATTGTCATTAATTTTAGTTCTGAAAAAGGCCT
ATTACTAAGATAAAAGCACTTCCTTCTGATGATAGCTAATTCACAAATTTA
CCTGGACAGCAAATTTGTTCACTAACCATTCCAGGATGGCCAATAAAATT
AATTTTGTAAACTTGCCAGTAAAAACTAAAGCTCAAATTCATTTTGGCTAC
AAGTTTACTTTCAGGGAATTGAGACTTTAATCCTTAACTGGCCAGTTTGCT
GACAGCAACCTTTTCCTAAGTTAATCAGAGGCCAGAGGATCAGTTAAAAT
CAATTCTACTTTTTGGCAGATGCAGATTAATGTCTTAATGTAGTCTGAACT
CACACTTTTCACTCTGGAGTGAGTTAAGGCGGGGCTTATAGAGAGCCAGG
GGATGGACTGACAAAACCCAGGAAGGATGGTCCACGCCTCACACTGCTGG
TCAAGGGCTCCATCCGCAAGCCTGCATGGTACATCTGTGGATGGGAGAGC
TTGATGCCCGGAGCCAGAGCTCCAGGCTGCGTAGGGGCTAAGAGCTCATG
GTACGGAGTTTTCTGATGGATACCAGGTGCTGTTTAACACAGGACTTTCTC
TGTCCATAAACACATTAGTCATTATACAAAAGAGTCATCTGAAGAATTTA
AAAATGACCAAATGGAAATATAAATATAAAATATCAAAAAATATGTCTAA
TAGTTTACTTGTGCAGTGATGTCCCAGCCTTCCTCCAAACAGACCAAAACT
GAGGAACCATTCTCAAGTACTTCTGATACAACCACAGCCAGCTGCATTAT
CCTGTGAAGCTAAGAGACAGGAAGGAGAACACAATTAGACCAAGCGCTT
TCTGAGTGCAGAAGTCAGAGAGAGTTGCTTTCAACCCTGGATAATCAAAC
GACTTAGGGATAAAGCGCTTGCACAAAAGAACCACATAATGTTTTAAATA
CCTAAAGTTCTTTCAAATCAAGATTAAACAATGACACTTTTGACTGGATGC
AATGACTCATGCCCATAATCCCAGCACTTTGGGAGGCCGAGCCGGGCAGG
TCATTTGAGGTTCGGAGTTCGAGACCAGCCTGGGCAACATAAGCAAGACC
CTGTCTCGAAAAATAAAAATAAAAAAGATACTCCTATTTGTCTATACTGT
GTACTAAATGAGAAAAAAACTTTCAATATATTTGTGCTAAGTTTTTAAAGC
CACATCAATTGGCTCAAAGTCCTTAATAAACAATTAAGGTATGAGAATAG
TATATAGATTTTAAAACCAGATGTATAACATTGTAATTTTAAAAAATATTT
TAATGATCATCAGACATAATTTGCAATTTGAATCCAACTCTGAACAAGAA
CAAAAATGAAAAAACCCATCTCTTAAACAGAGCTAGGCTACTTTAAACAC
AGGAATAAGGCAAATGCCCTACTCAGTAATGAGAAGCTCCTCATGTAACT
GTTTCTACAACATAGCTATCACCAGGCCTGAATTCTTTTGGGCTCAGAATT
TGTTCTAAGAAATGGCTATTATTAAAAAGTCAAAAAATAACAGATGCTGG
CGAGCCTGTGGAGAAAAGGGAATGTATATACATTTCTGGTGGGAATGTAA
ATTAGTTCAGCCATTGTAGAAAGTAGTTTGGTGATTTCTCAAATAACTTAA
AACAGAATTACCATTTGACCTAGCAATCCCATTACTGAGTATATACCCAA
AGGAATATAAATTGTTCTACCATAAAGTCACATGCACACACATGTTCAGT
GCTGTACTATTCACAATAGCAAAGACATGGAGTCAACCTATATGCCCATC
AACGGTAGACTGGATAAAGAAAATATGGTACATATACACCATGGAATACT

ACACAGCCATAAAAAGAATGAGCTTATGTCCTTTGCAGTAACACAGCTGA
AGCAAACTAACACAGGAATGGAAAACCAAATACTGCATATTCTCACTTAT
AAGTGGGAGCTAAACATTGAGTACACATGGACACAAAGAAAGGAATAGA
CGCCTGGGGCGTATTTGAGGTTGGAGGTGAGAGGAGGGTGAGGATAGAA
AAACTACCTATCAGGTACTATGCTTATTACCTGGGTGATGAAATAATCTGT
ACACCAAACTCCCAAGATATGCAATGTACCTATACAACAAACCTACAT

SBF2-AS1-MUT1, **binding sites of miR-338-3p were deletion-mutated**
CAGGTTCCAGCCCCGACCCGGGCGCGGGGCCGACTAGGGTCGGGTCCA
GTGTGCGGTGGTCGCTCCGCTCCGGGCCGCTCCGCTCTGGGCGTCAGGGC
GCGGGGAGCTGCCCCGGGGTTCTGTCCACCGGGGAGGAAAGCCACGAGC
ACTGAGCGCCTCCTGAGAGCCAGCCCTGACGTGAACTCATTTTATCTGCCA
CGACCCAGAAGGAGTCTACTGCTAAGATTTCAGCATGTCCTGTGGCTGAG
TTAATCAGAGTTATGACAGGAAGGTACCGGGCACACCATCGCAATGCTCC
ATCAATGCTAGTATGTTGTGTTCTTTCCTTCATATCAAGTCAACTCAAGCT
TGCTCTACTTACCTGGTGTACACAGTCTAAGAACTGTAAGAAGACTGGAG
CAAAACCACTCCCCTGACAGTTGAGGGTCAAGCTGCTCCTCTGACTGAAT
TTGTGACCAAAAGAGAGCCACTCTTTTTCAACCAACATCTGGAAGCCTTC
AAGTGTCCTATAAAAGGGATCACTGAGTAACTGAACCAGGGATGTCACCT
AGGGCATAAGCAGGATGGATTGTCATTAATTTTAGTTCTGAAAAAGGCCT
ATTACTAAGATAAAAGCACTTCCTTCTGATGATAGCTAATTCACAAATTTA
CCTGGACAGCAAATTTGTTCACTAACCATTCCAGGATGGCCAATAAAATT
AATTTTGTAAACTTGCCAGTAAAAACTAAAGCTCAAATTCATTTTGGCTAC
AAGTTTACTTTCAGGGAATTGAGACTTTAATCCTTAACTGGCCAGTTTGCT
GACAGCAACCTTTTCCTAAGTTAATCAGAGGCCAGAGGATCAGTTAAAAT
CAATTCTACTTTTTGGCAGATGCAGATTAATGTCTTAATGTAGTCTGAACT
CACACTTTTCACTCTGGAGTGAGTTAAGGCGGGGCTTATAGAGAGCCAGG
GGATGGACTGACAAAACCCAGGAAGGATGGTCCACGCCTCACACTCAAG
GGCTCCATCCGCAAGCCTGCATGGTACATCTGTGGATGGGAGAGCTTGAT
GCCCGGAGCCAGAGCTCCAGGCTGCGTAGGGGCTAAGAGCTCATGGTACG
GAGTTTTCTGATGGATACCAGGTGCTGTTTAACACAGGACTTTCTCTGTCC
ATAAACACATTAGTCATTATACAAAAGAGTCATCTGAAGAATTTAAAAAT
GACCAAATGGAAATATAAATATAAAATATCAAAAAATATGTCTAATAGTT
TACTTGTGCAGTGATGTCCCAGCCTTCCTCCAAACAGACCAAAACTGAGG
AACCATTCTCAAGTACTTCTGATACAACCACAGCCAGCTGCATTATCCTGT
GAAGCTAAGAGACAGGAAGGAGAACACAATTAGACCAAGCGCTTTCTGA
GTGCAGAAGTCAGAGAGAGTTGCTTTCAACCCTGGATAATCAAACGACTT
AGGGATAAAGCGCTTGCACAAAAGAACCACATAATGTTTTAAATACCTAA
AGTTCTTTCAAATCAAGATTAAACAATGACACTTTTGACTGGATGCAATG
ACTCATGCCCATAATCCCAGCACTTTGGGAGGCCGAGCCGGGCAGGTCAT
TTGAGGTTCGGAGTTCGAGACCAGCCTGGGCAACATAAGCAAGACCCTGT
CTCGAAAAATAAAAATAAAAAAGATACTCCTATTTGTCTATACTGTGTAC
TAAATGAGAAAAAAACTTTCAATATATTTGTGCTAAGTTTTTAAAGCCAC

ATCAATTGGCTCAAAGTCCTTAATAAACAATTAAGGTATGAGAATAGTAT
ATAGATTTTAAAACCAGATGTATAACATTGTAATTTTAAAAAATATTTTAA
TGATCATCAGACATAATTTGCAATTTGAATCCAACTCTGAACAAGAACAA
AAATGAAAAAACCCATCTCTTAAACAGAGCTAGGCTACTTTAAACACAGG
AATAAGGCAAATGCCCTACTCAGTAATGAGAAGCTCCTCATGTAACTGTT
TCTACAACATAGCTATCACCAGGCCTGAATTCTTTTGGGCTCAGAATTTGT
TCTAAGAAATGGCTATTATTAAAAAGTCAAAAAATAACACGAGCCTGTGG
AGAAAAGGGAATGTATATACATTTCTGGTGGGAATGTAAATTAGTTCAGC
CATTGTAGAAAGTAGTTTGGTGATTTCTCAAATAACTTAAAACAGAATTA
CCATTTGACCTAGCAATCCCATTACTGAGTATATACCCAAAGGAATATAA
ATTGTTCTACCATAAAGTCACATGCACACACATGTTCAGTGCTGTACTATT
CACAATAGCAAAGACATGGAGTCAACCTATATGCCCATCAACGGTAGACT
GGATAAAGAAAATATGGTACATATACACCATGGAATACTACACAGCCATA
AAAAGAATGAGCTTATGTCCTTTGCAGTAACACAGCTGAAGCAAACTAAC
ACAGGAATGGAAAACCAAATACTGCATATTCTCACTTATAAGTGGGAGCT
AAACATTGAGTACACATGGACACAAAGAAAGGAATAGACGCCTGGGGCG
TATTTGAGGTTGGAGGTGAGAGGAGGGTGAGGATAGAAAAACTACCTATC
AGGTACTATGCTTATTACCTGGGTGATGAAATAATCTGTACACCAAACTCC
CAAGATATGCAATGTACCTATACAACAAACCTACAT

SBF2-AS1-MUT2, **binding site of miR-362-3p was deletion-mutated**
CAGGTTCCAGCCCCGACCCGGGCGCGGGGCCGACTAGGGTCGGGTCCA
GTGTGCGGTGGTCGCTCCGCTCCGGGCCGCTCCGCTCTGGGCGTCAGGGC
GCGGGGAGCTGCCCCGGGGTTCTGTCCACCGGGGAGGAAAGCCACGAGC
ACTGAGCGCCTCCTGAGAGCCAGCCCTGACGTGAACTCATTTTATCTGCCA
CGACCCAGAAGGAGTCTACTGCTAAGATTTCAGCATGTCCTGTGGCTGAG
TTAATCAGAGTTATGACAGGAAGGTACCGGGCACACCATCGCAATGCTCC
ATCAATGCTAGTATGTTGTGTTCTTTCCTTCATATCAAGTCAACTCAAGCT
TGCTCTACTTACCTGGTGTACACAGTCTAAGAACTGTAAGAAGACTGGAG
CAAAACCACTCCCCTGACAGTTGAGGGTCAAGCTGCTCCTCTGACTGAAT
TTGTGACCAAAAGAGAGCCACTCTTTTTCAACCAACATCTGGAAGCCTTC
AAGTGTCCTATAAAAGGGATCACTGAGTAACTGAACCAGGGATGTCACCT
AGGGCATAAGCAGGATGGATTGTCATTAATTTTAGTTCTGAAAAAGGCCT
ATTACTAAGATAAAAGCACTTCCTTCTGATGATAGCTAATTCACAAATTTA
CCTGGACAGCAAATTTGTTCACTAACCATTCCAGGATGGCCAATAAAATT
AATTTTGTAAACTTGCCAGTAAAAACTAAAGCTCAAATTCATTTTGGCTAC
AAGTTTACTTTCAGGGAATTGAGACTTTAATCCTTAACTGGCCAGTTTGCT
GACAGCAACCTTTTCCTAAGTTAATCAGAGGCCAGAGGATCAGTTAAAAT
CAATTCTACTTTTTGGCAGATGCAGATTAATGTCTTAATGTAGTCTGAACT
CACACTTTTCACTCTGGAGTGAGTTAAGGCGGGGCTTATAGAGAGCCAGG
GGATGGACTGACAAAACCCAGGAAGGATGGTCCACGCCTCACACTGCTGG
TCAAGGGCTCCATCCGCAAGCCTGCATGGTACATCTGTGGATGGGAGAGC
TTGATGCCCGGAGCCAGAGCTCCAGGCTGCGTAGGGGCTAAGAGCTCATG

GTACGGAGTTTTCTGATGGATACCAGGTGCTGTTTAACACAGGACTTTCTC
TGTCCATAAACACATTAGTCATTATACAAAAGAGTCATCTGAAGAATTTA
AAAATGACCAAATGGAAATATAAATATAAAATATCAAAAAATATGTCTAA
TAGTTTACTTGTGCAGTGATGTCCCAGCCTTCCTCCAAACAGACCAAAACT
GAGGAACCATTCTCAAGTACTTCTGATACAACCACAGCCAGCTGCATTAT
CCTGTGAAGCTAAGAGACAGGAAGGAGAACACAATTAGACCAAGCGCTT
TCTGAGTGCAGAAGTCAGAGAGAGTTGCTTTCAACCCTGGATAATCAAAC
GACTTAGGGATAAAGCGCTTGCACAAAAGAACCACATAATGTTTTAAATA
CCTAAAGTTCTTTCAAATCAAGATTAAACAATGACACTTTTGACTGGATGC
AATGACTCATGCCCATAATCCCAGCACTTTGGGAGGCCGAGCCGGGCAGG
TCATTTGAGGTTCGGAGTTCGAGACCAGCCTGGGCAACATAAGCAAGACC
CTGTCTCGAAAAATAAAAATAAAAAAGATACTCCTATTTGTCTATACCTA
AATGAGAAAAAAACTTTCAATATATTTGTGCTAAGTTTTTAAAGCCACATC
AATTGGCTCAAAGTCCTTAATAAACAATTAAGGTATGAGAATAGTATATA
GATTTTAAAACCAGATGTATAACATTGTAATTTTAAAAAATATTTTAATGA
TCATCAGACATAATTTGCAATTTGAATCCAACTCTGAACAAGAACAAAAA
TGAAAAAACCCATCTCTTAAACAGAGCTAGGCTACTTTAAACACAGGAAT
AAGGCAAATGCCCTACTCAGTAATGAGAAGCTCCTCATGTAACTGTTTCT
ACAACATAGCTATCACCAGGCCTGAATTCTTTTGGGCTCAGAATTTGTTCT
AAGAAATGGCTATTATTAAAAAGTCAAAAAATAACAGATGCTGGCGAGC
CTGTGGAGAAAAGGGAATGTATATACATTTCTGGTGGGAATGTAAATTAG
TTCAGCCATTGTAGAAAGTAGTTTGGTGATTTCTCAAATAACTTAAAACAG
AATTACCATTTGACCTAGCAATCCCATTACTGAGTATATACCCAAAGGAA
TATAAATTGTTCTACCATAAAGTCACATGCACACACATGTTCAGTGCTGTA
CTATTCACAATAGCAAAGACATGGAGTCAACCTATATGCCCATCAACGGT
AGACTGGATAAAGAAAATATGGTACATATACACCATGGAATACTACACAG
CCATAAAAAGAATGAGCTTATGTCCTTTGCAGTAACACAGCTGAAGCAAA
CTAACACAGGAATGGAAAACCAAATACTGCATATTCTCACTTATAAGTGG
GAGCTAAACATTGAGTACACATGGACACAAAGAAAGGAATAGACGCCTG
GGGCGTATTTGAGGTTGGAGGTGAGAGGAGGGTGAGGATAGAAAAACTA
CCTATCAGGTACTATGCTTATTACCTGGGTGATGAAATAATCTGTACACCA
AACTCCCAAGATATGCAATGTACCTATACAACAAACCTACAT

SBF2-AS1-MUT, **binding sites of miR-338-3p and miR-362-3p were deletion-mutated**

CAGGTTCCAGCCCCGACCCGGGCGCGGGGCCGACTAGGGTCGGGTCCA
GTGTGCGGTGGTCGCTCCGCTCCGGGCCGCTCCGCTCTGGGCGTCAGGGC
GCGGGGAGCTGCCCCGGGGTTCTGTCCACCGGGGAGGAAAGCCACGAGC
ACTGAGCGCCTCCTGAGAGCCAGCCCTGACGTGAACTCATTTTATCTGCCA
CGACCCAGAAGGAGTCTACTGCTAAGATTTCAGCATGTCCTGTGGCTGAGT

TAATCAGAGTTATGACAGGAAGGTACCGGGCACACCATCGCAATGCTCCAT
CAATGCTAGTATGTTGTGTTCTTTCCTTCATATCAAGTCAACTCAAGCTTGC
TCTACTTACCTGGTGTACACAGTCTAAGAACTGTAAGAAGACTGGAGCAAA
ACCACTCCCCTGACAGTTGAGGGTCAAGCTGCTCCTCTGACTGAATTTGTG
ACCAAAAGAGAGCCACTCTTTTTCAACCAACATCTGGAAGCCTTCAAGTG
TCCTATAAAAGGGATCACTGAGTAACTGAACCAGGGATGTCACCTAGGGCA
TAAGCAGGATGGATTGTCATTAATTTTAGTTCTGAAAAAGGCCTATTACTAA
GATAAAAGCACTTCCTTCTGATGATAGCTAATTCACAAATTTACCTGGACAG
CAAATTTGTTCACTAACCATTCCAGGATGGCCAATAAAATTAATTTTGTAAA
CTTGCCAGTAAAAACTAAAGCTCAAATTCATTTTGGCTACAAGTTTACTTTC
AGGGAATTGAGACTTTAATCCTTAACTGGCCAGTTTGCTGACAGCAACCTT
TTCCTAAGTTAATCAGAGGCCAGAGGATCAGTTAAAATCAATTCTACTTTTT
GGCAGATGCAGATTAATGTCTTAATGTAGTCTGAACTCACACTTTTCACTCT
GGAGTGAGTTAAGGCGGGGCTTATAGAGAGCCAGGGGATGGACTGACAAA
ACCCAGGAAGGATGGTCCACGCCTCACACTCAAGGGCTCCATCCGCAAGC
CTGCATGGTACATCTGTGGATGGGAGAGCTTGATGCCCGGAGCCAGAGCTC
CAGGCTGCGTAGGGGCTAAGAGCTCATGGTACGGAGTTTTCTGATGGATAC
CAGGTGCTGTTTAACACAGGACTTTCTCTGTCCATAAACACATTAGTCATTA
TACAAAAGAGTCATCTGAAGAATTTAAAAATGACCAAATGGAAATATAAAT
ATAAAATATCAAAAAATATGTCTAATAGTTTACTTGTGCAGTGATGTCCCAG
CCTTCCTCCAAACAGACCAAAACTGAGGAACCATTCTCAAGTACTTCTGAT
ACAACCACAGCCAGCTGCATTATCCTGTGAAGCTAAGAGACAGGAAGGAG
AACACAATTAGACCAAGCGCTTTCTGAGTGCAGAAGTCAGAGAGAGTTGC
TTTCAACCCTGGATAATCAAACGACTTAGGGATAAAGCGCTTGCACAAAAG
AACCACATAATGTTTTAAATACCTAAAGTTCTTTCAAATCAAGATTAAACAA
TGACACTTTTGACTGGATGCAATGACTCATGCCCATAATCCCAGCACTTTGG
GAGGCCGAGCCGGGCAGGTCATTTGAGGTTCGGAGTTCGAGACCAGCCTG
GGCAACATAAGCAAGACCCTGTCTCGAAAAATAAAAATAAAAAAGATACT
CCTATTTGTCTATACCTAAATGAGAAAAAAACTTTCAATATATTTGTGCTAAG
TTTTTAAAGCCACATCAATTGGCTCAAAGTCCTTAATAAACAATTAAGGTAT
GAGAATAGTATATAGATTTTAAAACCAGATGTATAACATTGTAATTTTAAAAA
ATATTTTAATGATCATCAGACATAATTTGCAATTTGAATCCAACTCTGAACAA
GAACAAAAATGAAAAAACCCATCTCTTAAACAGAGCTAGGCTACTTTAAAC
ACAGGAATAAGGCAAATGCCCTACTCAGTAATGAGAAGCTCCTCATGTAAC
TGTTTCTACAACATAGCTATCACCAGGCCTGAATTCTTTTGGGCTCAGAATT
TGTTCTAAGAAATGGCTATTATTAAAAAGTCAAAAAATAACACGAGCCTGT
GGAGAAAAGGGAATGTATATACATTTCTGGTGGGAATGTAAATTAGTTCAG
CCATTGTAGAAAGTAGTTTGGTGATTTCTCAAATAACTTAAAACAGAATTAC
CATTTGACCTAGCAATCCCATTACTGAGTATATACCCAAAGGAATATAAATTG
TTCTACCATAAAGTCACATGCACACACATGTTCAGTGCTGTACTATTCACAA
TAGCAAAGACATGGAGTCAACCTATATGCCCATCAACGGTAGACTGGATAA
AGAAAATATGGTACATATACACCATGGAATACTACACAGCCATAAAAAGAAT
GAGCTTATGTCCTTTGCAGTAACACAGCTGAAGCAAACTAACACAGGAATG
GAAAACCAAATACTGCATATTCTCACTTATAAGTGGGAGCTAAACATTGAGT

ACACATGGACACAAAGAAAGGAATAGACGCCTGGGGCGTATTTGAGGTTG
GAGGTGAGAGGAGGGTGAGGATAGAAAAACTACCTATCAGGTACTATGCTT
ATTACCTGGGTGATGAAATAATCTGTACACCAAACTCCCAAGATATGCAATG
TACCTATACAACAAACCTACAT

Figure S1: Screening early stage-specific lncRNAs. 5 outliers were excluded from hierarchical clustering of cancer tissue samples (A). 12 significant co-expression gene modules across all 508 sampling sets were detected with WGCNAs (B). Soft thresholding power to achieve scale-free topology ($\beta = 2$) (C). The correlation within gene sets of module greenyellow (D). The heatmap of all 3250 differentially expressed genes in GSE19804 (E).

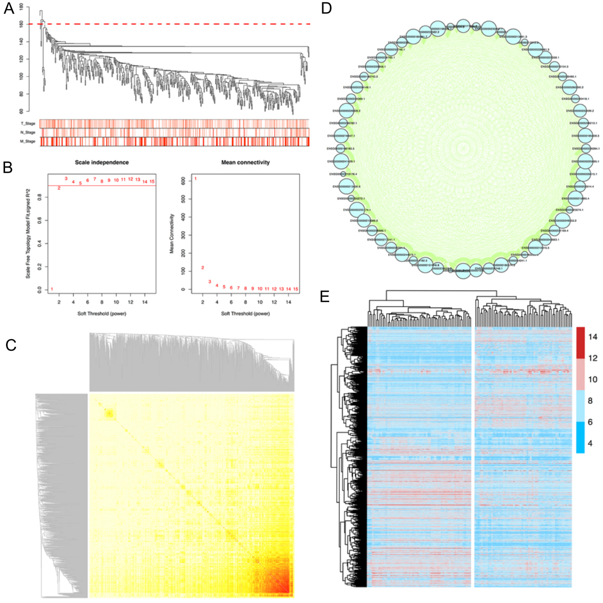
A
T_Stage
N_Stage
M_Stage
B
Scale independence
Mean connectivity
Scale Free Topology Model Fit,signed R^2
Mean Connectivity
Soft Threshold (power)
Soft Threshold (power)
C
D
E
14
12
10
8
6
4

Figure S2. SBF2-AS1promotes H1299 cell proliferation. Cell cycle was arrested at G1 phase in H1299 cells upon SBF2-AS1 knockdown (A). Expression of Cyclin D1 and P21 after ectopic expression and silence of SBF2-AS1 in H1299 cells (B). CCK8 assay (C), EdU (D), and colony formation assay (E) in H1299 cells after ectopic and silence of SBF2-AS1

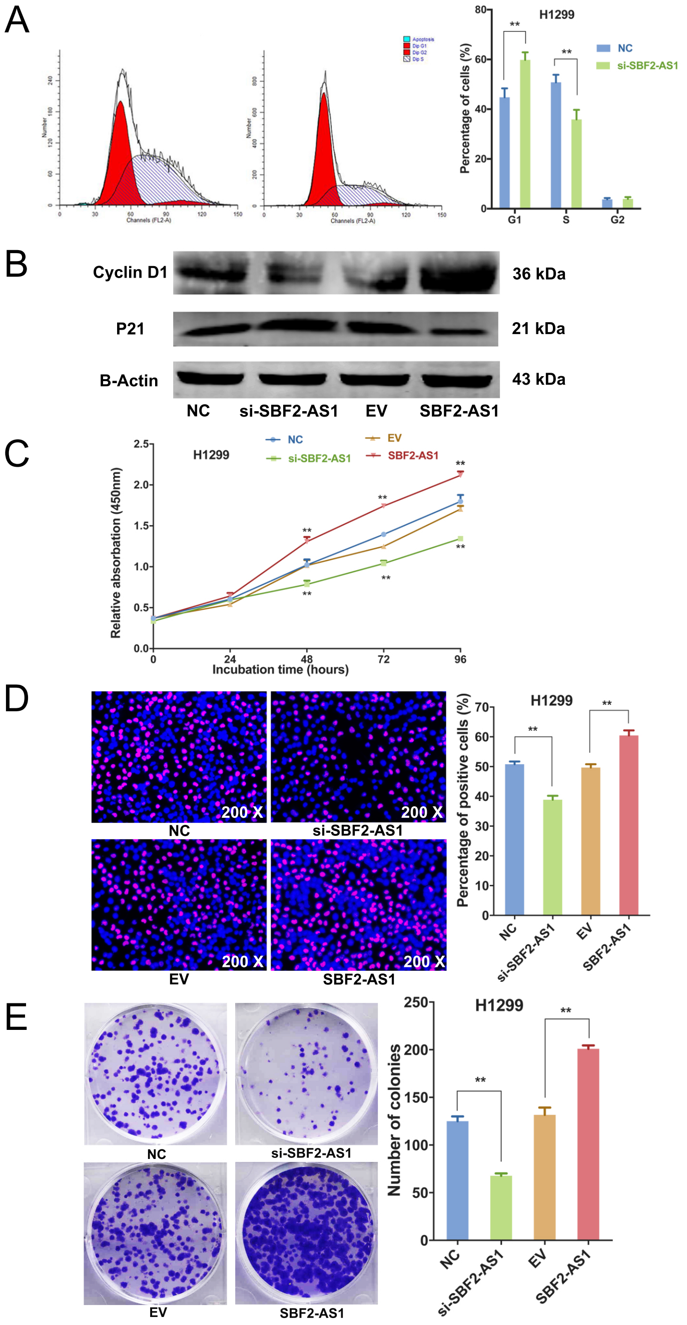

A
Apoptosis
Dip G1
Dip G2
Dip S
Number
Channels (FL2-A)
H1299
NC
si-SBF2-AS1
Percentage of cells (%)
G1
S
G2
B
Cyclin D1
36 kDa
P21
21 kDa
B-Actin
43 kDa
NC
si-SBF2-AS1
EV
SBF2-AS1
C
H1299
NC
si-SBF2-AS1
EV
SBF2-AS1
Relative absorbation (450nm)
Incubation time (hours)
D
200 X
NC
si-SBF2-AS1
EV
SBF2-AS1
H1299
Percentage of positive cells (%)
E
NC
si-SBF2-AS1
EV
SBF2-AS1
H1299
Number of colonies

Figure S3. Colony formation (A) and EdU (B) assay in H1299 cells. CCK8 assay in A549 (C) and H1299 (D) cells.

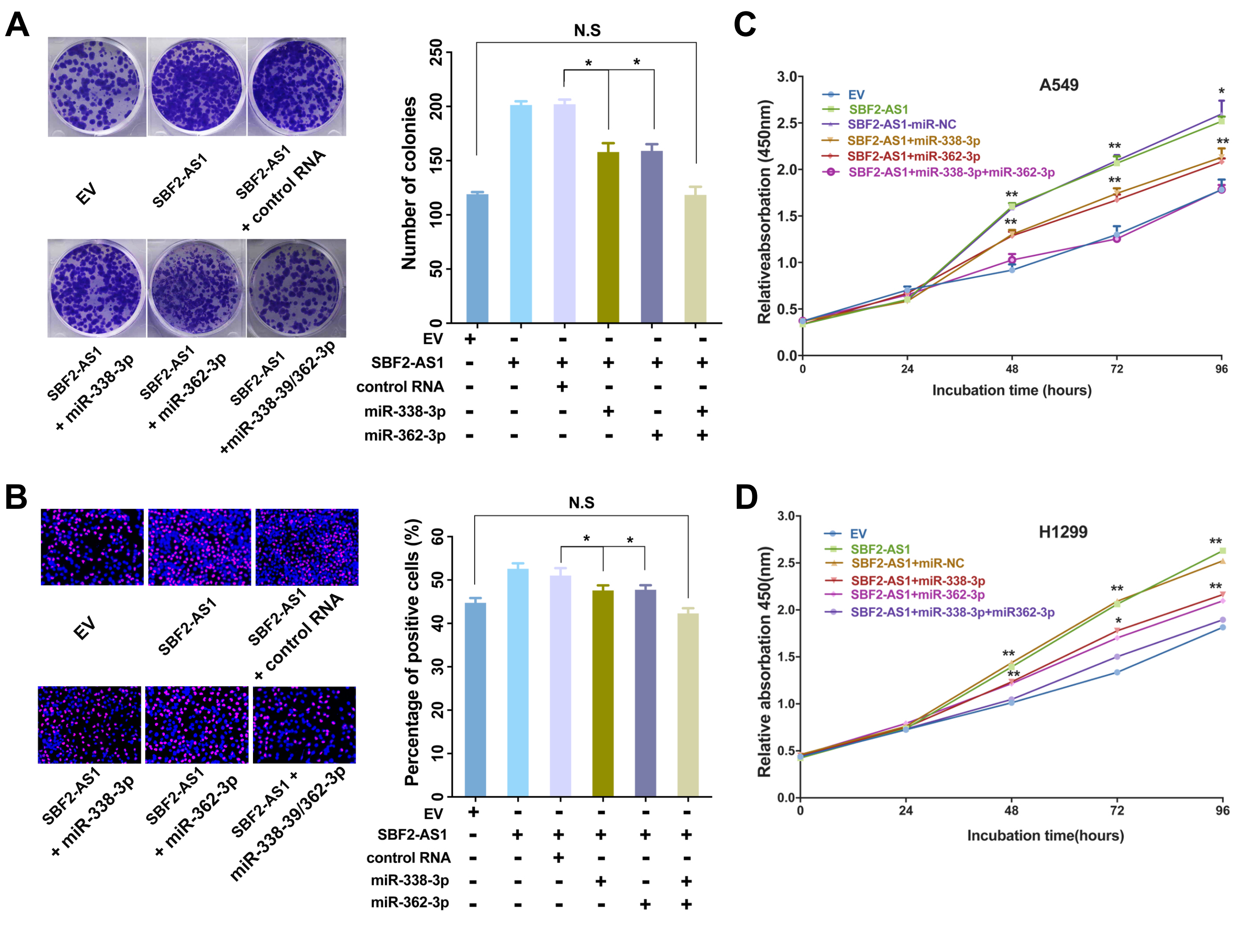
A
EV
SBF2-AS1
SBF2-AS1 + control RNA
SBF2-AS1 + miR-338-3p
SBF2-AS1 + miR-362-3p
SBF2-AS1 +miR-338-39/362-3p
N.S
Number of colonies
EV + - - - - -
SBF2-AS1 - + + + + +
control RNA - - + - - -
miR-338-3p - - - + - +
miR-362-3p - - - - + +
B
EV
SBF2-AS1
SBF2-AS1 + control RNA
SBF2-AS1 + miR-338-3p
SBF2-AS1 + miR-362-3p
SBF2-AS1 + miR-338-39/362-3p
N.S
Percentage of positive cells (%)
EV + - - - - -
SBF2-AS1 - + + + + +
control RNA - - + - - -
miR-338-3p - - - + - +
miR-362-3p - - - - + +
C
A549
EV
SBF2-AS1
SBF2-AS1-miR-NC
SBF2-AS1+miR-338-3p
SBF2-AS1+miR-362-3p
SBF2-AS1+miR-338-3p+miR-362-3p
Relativeabsorbation (450nm)
Incubation time (hours)
D
H1299
EV
SBF2-AS1
SBF2-AS1+miR-NC
SBF2-AS1+miR-338-3p
SBF2-AS1+miR-362-3p
SBF2-AS1+miR-338-3p+miR362-3p
Relative absorbation 450(nm)
Incubation time(hours)

Figure S4. Venn plot of candidate target genes (A). ceRNA network driven by SBF2-AS1 (B).

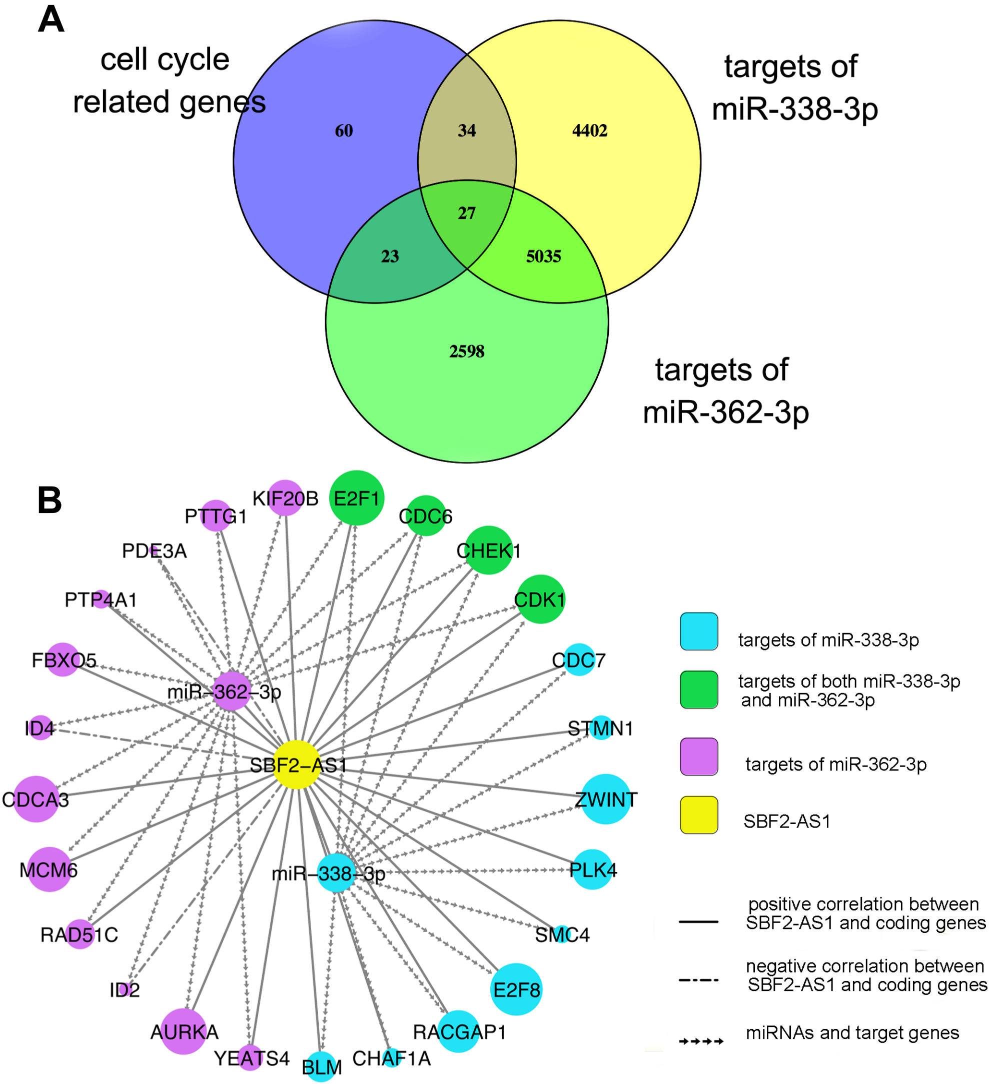
A
cell cycle related genes
targets of miR-338-3p
targets of miR-362-3p
60
34
4402
27
23
5035
2598
B
SBF2–AS1
miR–362–3p
miR–338–3p
PTTG1
KIF20B
E2F1
CDC6
CHEK1
CDK1
PDE3A
PTP4A1
FBXO5
ID4
CDCA3
MCM6
RAD51C
ID2
AURKA
YEATS4
BLM
CHAF1A
RACGAP1
E2F8
SMC4
PLK4
ZWINT
STMN1
CDC7
targets of miR-338-3p
targets of both miR-338-3p and miR-362-3p
targets of miR-362-3p
SBF2-AS1
positive correlation between SBF2-AS1 and coding genes
negative correlation between SBF2-AS1 and coding genes
miRNAs and target genes
