# Supplementary material for: Long Noncoding RNA SBF2-AS1 Is Critical for Tumorigenesis of Early-Stage Lung Adenocarcinoma
Source: Mol Ther Nucleic Acids. 2019 Apr 13;16:543–53. doi: 10.1016/j.omtn.2019.04.004 (PMC6506611; doi:10.1016/j.omtn.2019.04.004)
Supplement: Document S3. Article plus Supplemental Information [file mmc3.pdf]

Molecular Therapy
Nucleic Acids
Original Article

Long Noncoding RNA SBF2-AS1 Is Critical for Tumorigenesis of Early-Stage Lung Adenocarcinoma

Rui Chen,[1,2,15] Wenjia Xia,[1,15] Siwei Wang,[1,15] Youtao Xu,[1] Zhifei Ma,[1] Weizhang Xu,[1] Erbao Zhang,[3] Jie Wang,[1,4] Tian Fang,[5] Quan'an Zhang,[6] Gaochao Dong,[1,5] William Chi-shing Cho,[7] Patrick C. Ma,[8] Giovanni Brandi,[9,10] Simona Tavolari,[9,10] Peter Ujhazy,[11] Giulio Metro,[12] Helmut H. Popper,[13] Rong Yin,[1] Mantang Qiu,[14] and Lin Xu[1]

[1]Department of Thoracic Surgery, Jiangsu Key Laboratory of Molecular and Translational Cancer Research, Jiangsu Cancer Hospital, Jiangsu Institute of Cancer Research, Nanjing Medical University Affiliated Cancer Hospital, Nanjing 210009, China; [2]Department of Cardiothoracic Surgery, Taixing People's Hospital, The Affiliated Taixing Hospital of Yangzhou University, Taixing 225400, China; [3]Department of Epidemiology and Biostatistics, Jiangsu Key Lab of Cancer Biomarkers, Prevention and Treatment, Collaborative Innovation Center for Cancer Personalized Medicine, School of Public Health, Nanjing Medical University, Nanjing 210009, China; [4]Department of Scientific Research, Jiangsu Cancer Hospital, Jiangsu Institute of Cancer Research, Nanjing Medical University Affiliated Cancer Hospital, Nanjing 210009, China; [5]Department of Comparative Medicine, Jingling Hospital, Nanjing University School of Medicine, Nanjing 210002, China; [6]Department of Oncology, Nanjing Medical University Affiliated Jiangning Hospital, Nanjing 211100, China; [7]Department of Clinical Oncology, Queen Elizabeth Hospital, Hong Kong, China; [8]Aerodigestive Oncology Translational Research THOR, Department of Solid Tumor Oncology, Taussig Cancer Institute, Cleveland Clinic, Cleveland, OH, USA; [9]Department of Experimental Diagnostic and Specialty Medicine, S. Orsola-Malpighi University Hospital, Bologna, Italy; [10]Center for Applied Biomedical Research, S. Orsola-Malpighi University Hospital, Bologna, Italy; [11]Translational Research Program, Division of Cancer Treatment and Diagnosis, National Cancer Institute, Bethesda, MD, USA; [12]Division of Medical Oncology, Santa Maria della Misericordia Hospital, Azienda Ospedaliera di Perugia, via Dottori, 106156 Perugia, Italy; [13]Department of Pathology, Medical University of Graz, Auenbruggerplatz 25, Graz 8036, Austria; [14]Department of Thoracic Surgery, Peking University People's Hospital, Beijing 100044, China

Emerging evidence demonstrates that long non-coding RNAs (lncRNAs) are deeply involved in the development of various cancers. This study identified that SBF2-AS1, an early-stage-specific lncRNA, is critical for the tumorigenesis of lung adenocarcinoma (LUAD). We first analyzed LUAD transcriptome data from The Cancer Genome Atlas and the GEO database by weighted gene co-expression network analysis (WGCNA). Five early LUAD-specific lncRNAs were filtered out, and only SBF2-AS1 was upregulated in LUAD. High expression of SBF2-AS1 indicates poor survival of LUAD, especially the early-stage LUAD, but not lung squamous cell carcinoma. SBF2-AS1 promotes LUAD cells proliferation *in vitro*, and RNA-sequencing data shows that many cell-cycle-related genes were downregulated after SBF2-AS1 knockdown. Mechanically, SBF2-AS1 could competitively bind with miR-338-3p and miR-362-3p to increase E2F1 expression. Finally, we show that the SBF2-AS1-miR-338-3p/362-3p-E2F1 axis could promote LUAD tumorigenesis *in vitro* and *in vivo*. Our study demonstrates that SBF2-AS1, an early-stage-specific lncRNA, promotes LUAD tumorigenesis by sponging miR-338-3p and miR-362-3p and increasing E2F1 expression. The SBF2-AS1-miR-338-3p/362-3p-E2F1 regulatory axis may serve as a prognostic marker and potential therapeutic target for LUAD.

INTRODUCTION

Lung cancer is the leading cause of cancer death worldwide.[1,2] During the past decades, the pathological constitution of lung cancer has gradually changed, and lung adenocarcinoma (LUAD) has become a most prevalent subtype, accounting for approximately 70% of total lung cancer, especially in Eastern Asia.[3,4] In China, recent data from a cohort of 21,113 lung cancer patients also showed that the proportion of early-stage LUAD, which featured solid or subsolid nodules, has dramatically increased, almost 5-fold, up to 30.54% in 2012, compared with 6.25% in 1999.[5] Therefore, in order to better understand how early-stage LUAD developed, it is helpful for us to focus on the tumorigenesis of LUAD and identify the underlying dominant forces for it.

The accumulation of genomic instability is one of the key events required for tumorigenesis.[6] With respect to LUAD, many well-known coding driver genes were identified, such as *EGFR*, *KRAS*, and *MYC*, as well as *PIK3CA*, which modulates numerous genetic regulatory mechanisms and forms a large network.[7] However, the

Received 20 July 2018; accepted 4 April 2019;
https://doi.org/10.1016/j.omtn.2019.04.004.

[15]These authors contributed equally to this work.

Correspondence: Lin Xu, Jiangsu Collaborative Innovation Center on Cancer Personalized Medicine, Nanjing Medical University, Nanjing 210009, China.
E-mail: xulin83cn@gmail.com
Correspondence: Mantang Qiu, Department of Thoracic Surgery, Peking University People's Hospital, 11 South Xizhimen Street, Beijing 100044, China.
E-mail: qiumantang@163.com
Correspondence: Rong Yin, Jiangsu Collaborative Innovation Center on Cancer Personalized Medicine, Nanjing Medical University, Nanjing 210009, China.
E-mail: yinhero001@126.com

This is an open access article under the CC BY license (http://creativecommons.org/licenses/by/4.0/).

molecular basis of LUAD tumorigenesis is far less understood. During the past decade, noncoding RNAs are drawing new attention,[6,8] especially the long noncoding RNAs (lncRNAs), which are a kind of RNA transcript longer than 200 nt without coding capacity.[9,10] According to the Encyclopedia of DNA Elements (ENCODE) project, more than 16,000 lncRNAs transcribed from the human genome were identified.[11] Mounting evidence has proved that lncRNAs play major roles in cancer.[12]

Several LUAD tumorigenesis-associated lncRNAs have been identified in recent years. For instance, a second messenger PIP3-binding lncRNA, LINK-A, has been demonstrated as upregulated in LUAD and could promote tumorigenesis via facilitating AKT-PIP3 interaction and consequent AKT hyperactivation.[13] In addition, an imprinted lncRNA, MEG3, is hypermethylated in LUAD, and its downregulation contributes to nickel-induced malignant transformation of human bronchial epithelial cells.[14] We previously identified that an oncogenic lncRNA, LUAD transcript 1 (LUADT1), could promote LUAD progression via epigenetically suppressing p27.[15] However, it remains a big challenge to effectively identify more critical and dominant lncRNAs from numerous lncRNA transcripts. Various bioinformatics tools have been developed to identify critical genes from high-throughput data. Among them, weighted gene co-expression network analysis (WGCNA) is a powerful tool for finding modules of highly correlated genes and has been extensively adopted to identify candidate biomarkers or therapeutic targets.[16]

In the present study, we identified tumor-stage-specific lncRNAs in LUAD by WGCNA from The Cancer Genome Atlas (TCGA) RNA-sequencing data. Eventually, we found that SBF2-AS1, a lncRNA upregulated in non-small-cell lung cancer that we previously reported,[17] is critical for the tumorigenesis of LUAD. Further experiments have confirmed that SBF2-AS1 could promote LUAD tumorigenesis by sponging miR-338/miR-362 and subsequent increased expression of E2F1.

RESULTS

Screening Early-Stage-Specific lncRNAs in LUAD

To identify lncRNAs involved in tumorigenesis of early-stage LUAD, we analyzed the HTSeq-Counts data of 515 LUAD cases from TCGA (for WGCNA) and an expression dataset of 60 pairs of early-stage LUAD specimens (GEO: GSE19804).[18] The overall data analysis workflow is shown in Figure 1A. First, 7,320 differentially expressed genes in LUAD were detected by DESeq2 from the TCGA dataset (Figure 1B). Then, WGCNA elucidated 13 co-expressed modules using 7,320 differentially expressed genes and 508 samples (outliers were excluded) (Figures 1C, 1D, and S1A). To determine whether any of the identified expression modules were associated with clinical stages, we calculated the Pearson's correlation coefficient (PCC) between the module eigengenes (MEs) and tumor lymph node matastasis (TNM) stages. "greenyellow" and "turquoise" were two modules with the highest correlation coefficients to T stages (Figure 1E). A total of 59 T-stage-specific lncRNAs were screened from the "greenyellow" and "turquoise" modules. The previously described dataset (GEO: GSE19804) served as a filter to identify early-stage-specific lncRNAs; and, finally, 5 lncRNAs (ENSG00000241684, ENSG00000278921, ENSG00000254109, ENSG00000246273, and ENSG00000180769) were selected from the aforementioned 59 lncRNAs (Figure 1E). Notably, ENSG00000246273 (official symbol: SBF2-AS1) was upregulated in LUAD in both datasets, while the other 4 lncRNAs were downregulated in LUAD. Therefore, SBF2-AS1 was selected for further investigation.

High SBF2-AS1 Expression Correlates with Poor Survival of LUAD but Not LUSC

To explore whether SBF2-AS1 is associated with the survival of lung cancer patients, we performed gene set enrichment analysis (GSEA) in a lung cancer dataset of 111 samples (GEO: GSE3141).[19] SBF2-AS1 expression is positively correlated with a gene set of poor survival (Figure 2A) while negatively correlated with a gene set of good survival (Figure 2B), indicating that SBF2-AS1 might be a biomarker of poor survival. SBF2-AS1 expression was then analyzed online (kmplot.com/analysis/), using microarray data from 673 LUAD and 271 lung squamous cell carcinoma (LUSC) patients,[20] and the results in Figure 2C show that high SBF2-AS1 expression is associated with poor overall survival in LUAD (hazard ratio [HR] = 1.38; 95% confidence interval [CI]: 1.07–1.73; log rank $p = 0.012$) but not with LUSC (HR = 0.78; log rank $p = 0.11$). Notably, given that SBF2-AS1 is an early-stage-specific lncRNA, when limited to stage T1 LUAD, we observed that patients with high SBF2-AS1 expression had significantly poorer survival than those with low SBF2-AS1 expression (HR = 2.58; 95% CI: 1.26–5.31; log rank $p = 0.0037$; Figure 2C). In addition, TCGA data (Figure 2D) also confirm that high SBF2-AS1 expression indicated shorter survival time in LUAD but not in LUSC (log rank $p = 0.04$ for LUAD, and log rank $p = 0.213$ for LUSC; data are from https://ibl.mdanderson.org/tanric/_design/basic/index.html). Together, these lines of evidence demonstrate that SBF2-AS1 could be a specific biomarker and a poor prognostic factor for LUAD.

SBF2-AS1 Promotes Proliferation of LUAD Cells via Regulating Cell Cycle

Due to the prognostic value of SBF2-AS1 in LUAD, we further explored its biological functions. Small interfering RNA (siRNA) and expression vector were utilized to knock down and overexpress SBF2-AS1, respectively. RNA sequencing was first performed to identify a gene expression profile after silence of SBF2-AS1 in A549 cells, and the results revealed that the expression of numerous genes was altered (Table S2). GSEAs suggested that these deregulated genes are mostly involved in biological processes of cell cycle and proliferation (Figure 3A; Table S3), indicating that SBF2-AS1 might mainly impact cell cycle and proliferation. As shown, knockdown of SBF2-AS1 led to G1 phase cell-cycle arrest both in A549 (Figures 3B and 3C) and H1299 cells (Figure S2A). Accordingly, typical cell-cycle markers such as cyclin D1 were upregulated, whereas p21 was downregulated during the ectopic expression of SBF2-AS1 (Figure 3C;

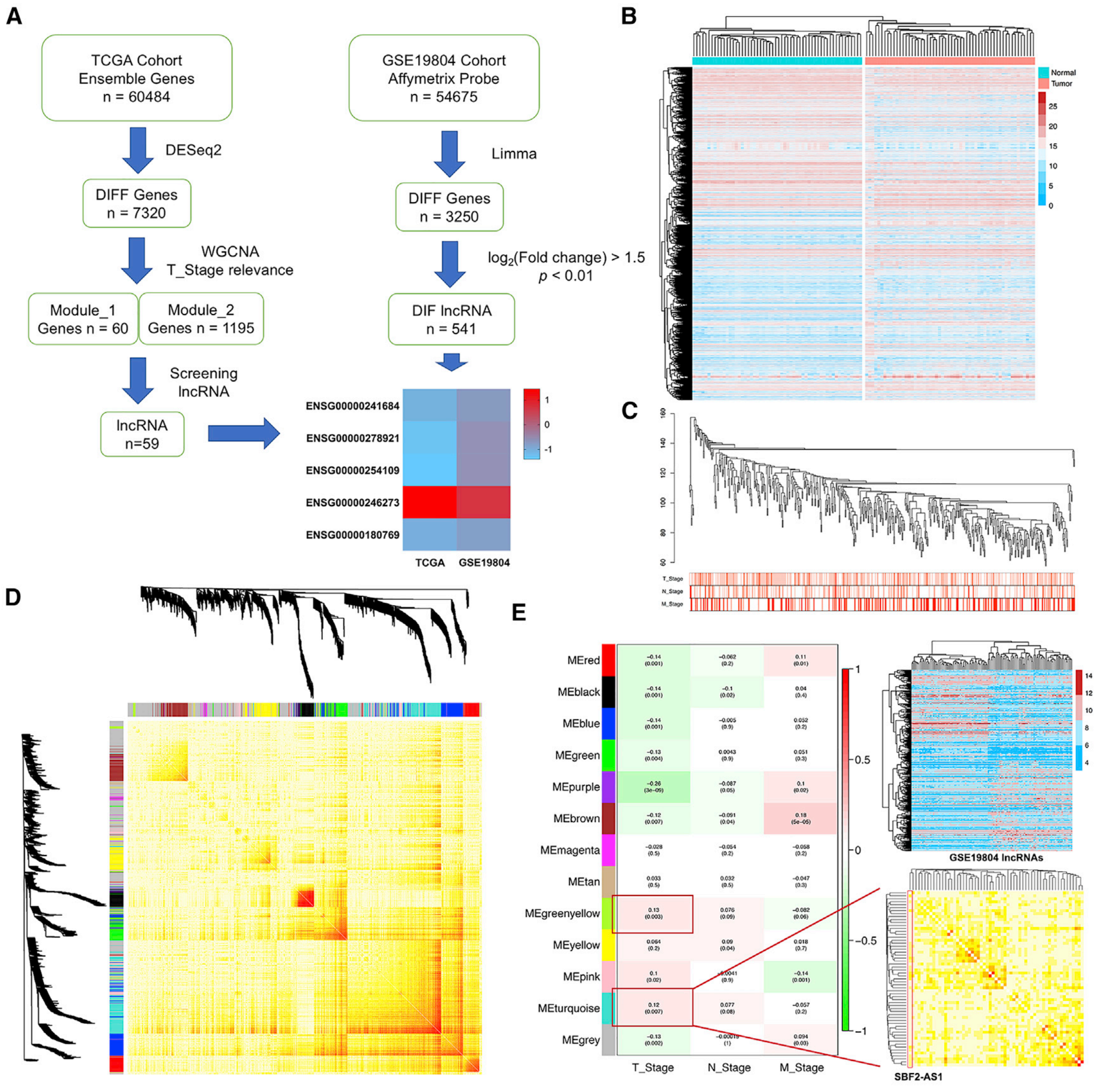

Figure 1. Screening Early-Stage-Specific lncRNAs in LUAD

The flowchart of WGCNAs and lncRNAs screening (A). 7,320 differentially expressed genes screened from the TCGA LUAD project (B). Hierarchical clustering of 508 LUAD cancer tissue samples based on 7,320 differentially expressed genes (C). 12 significant co-expression gene modules across all 508 sampling sets were demonstrated with WGCNA TOMplot. Identified co-expression modules were represented by color classifiers (gray is assigned to genes that are not part of any module) (D). PCC between the MEs and TNM stages. A positive value indicates that the genes within a particular co-expression module increase as the variable increases, and each PCC value is accompanied by the corresponding p value in brackets (left panel). The heatmap of all 541 differentially expressed lncRNAs in GSE19804 (top panel); screened 59 lncRNAs were subjected to the correlation heatmap (bottom panel) (E).

Figure S2B). Ectopic expression of SBF2-AS1 promoted LUAD cell proliferation, while knockdown of SBF2-AS1 inhibited cell proliferation ability, as revealed by the CCK-8 (Figure 3D) and 5-ethynyl-2'-deoxyuridine (EdU) incorporation assay (Figure 3E; Figures S2C and S2D). Both loss- and gain-of-function experiments showed that SBF2-AS1 could increase the colony formation ability of A549 (Figure 3F) and H1299 cells (Figure S2E). Collectively, our results demonstrate that SBF2-AS1 could promote cell cycling and cell proliferation in LUAD.

SBF2-AS1 Binds with miR-338-3p and miR-362-3p

Real-time PCR of fractionated nuclear and cytoplasmic RNA showed that SBF2-AS1 mainly localized in cytoplasm in LUAD cells (Figure 4A). Sub-cellular distribution suggests that SBF2-AS1 might have distinct a regulatory mechanism in cytoplasm. It has been proposed that RNA transcripts (mRNA, lncRNA, pesudogene, etc.) could cross-talk with each other using common microRNA (miRNA) binding sites, i.e., the competing endogenous RNA (ceRNA) hypothesis.[21,22] Thus, we hypothesized that

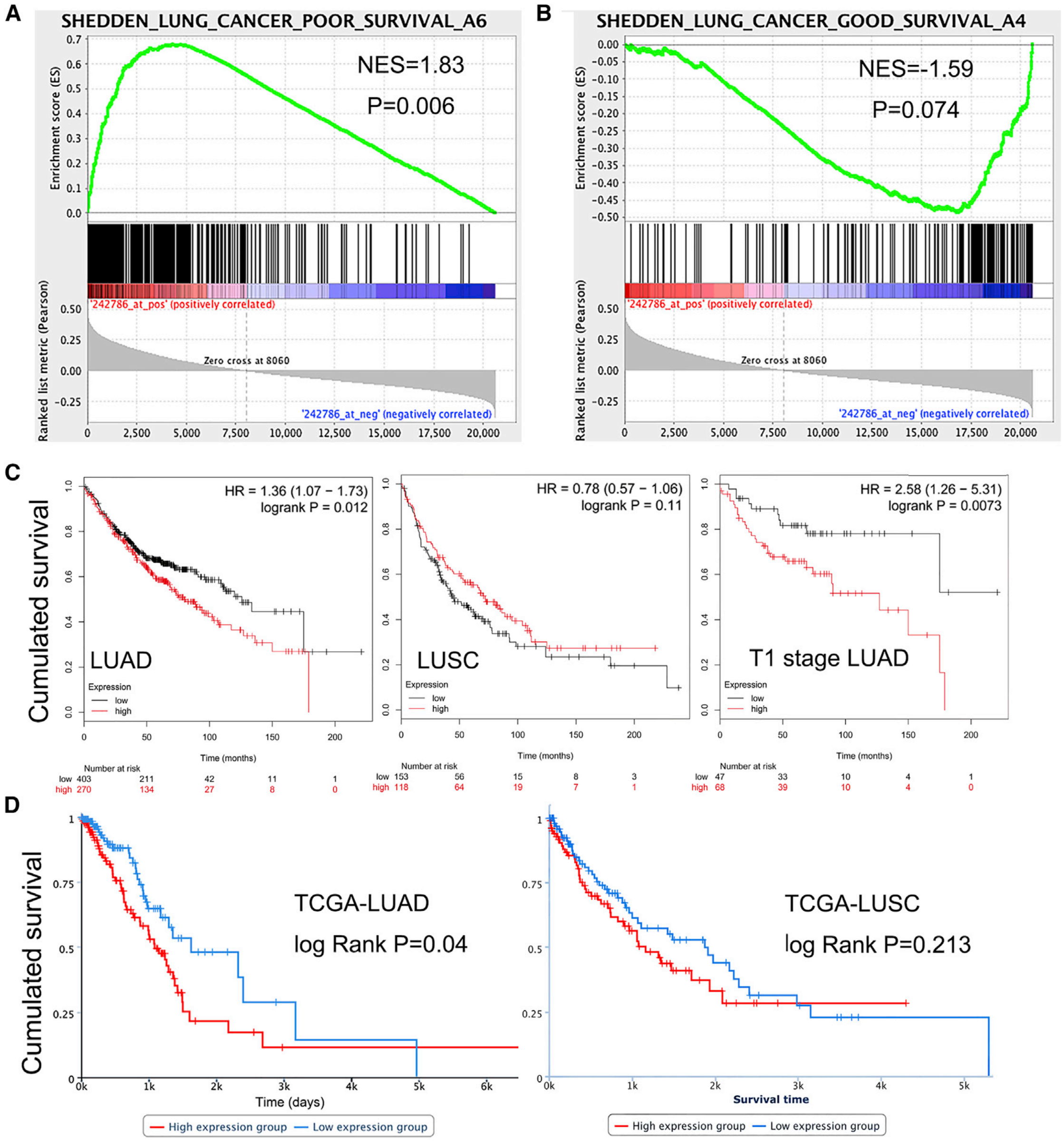

Figure 2. High Expression Indicates Poor Survival of LUAD

GSEA results showed that SBF2-AS1 was positively correlated with a gene set of poor survival (p = 0.006; A) and negatively correlated with a gene set of good survival (p = 0.074; B). High expression of SBF2-AS1 was associated with shorter overall survival time in LUAD, especially in T1-stage LUAD, but not in LUSC (C). TCGA data also suggest that high SBF2-AS1 expression associates with shorter survival time in LUAD but not in LUSC (D). NES, normalized enrichment score.

SBF2-AS1 may function in the ceRNA mechanism. The miRanda algorithm predicted that there were various miRNA binding sites within the SBF2-AS1 transcript (Table S4). Together with the photoactivatable ribonucleoside-enhanced crosslinking and immunoprecipitation (PAR-CLIP) sequencing data,[23,24] we identified 4 miRNAs that could potentially bind with SBF2-AS1: miR-338-3p, miR-362-3p (Figure 4B), miR-329, and miR-140. An RNA immunoprecipitation (RIP) assay demonstrated that SBF2-AS1 could bind with Ago2 protein in A549 cells (Figure 4C). To confirm the binding between SBF2-AS1 and miR-338-3p and miR-362-3p, we synthesized biotin-labeled miRNAs and performed a miRNA pull-down assay in A549 cells. Results suggested that miR-338-3p and miR-362-3p could significantly enrich SBF2-AS1 (Figure 4D), while miR-329 and miR-140 did not. In addition, overexpression of miR-338-3p and miR-362-3p did not alter SBF2-AS1 expression level (Figures 4E and 4F). These experiments prove that SBF2-AS1 could bind with miR-338-3p and miR-362-3p.

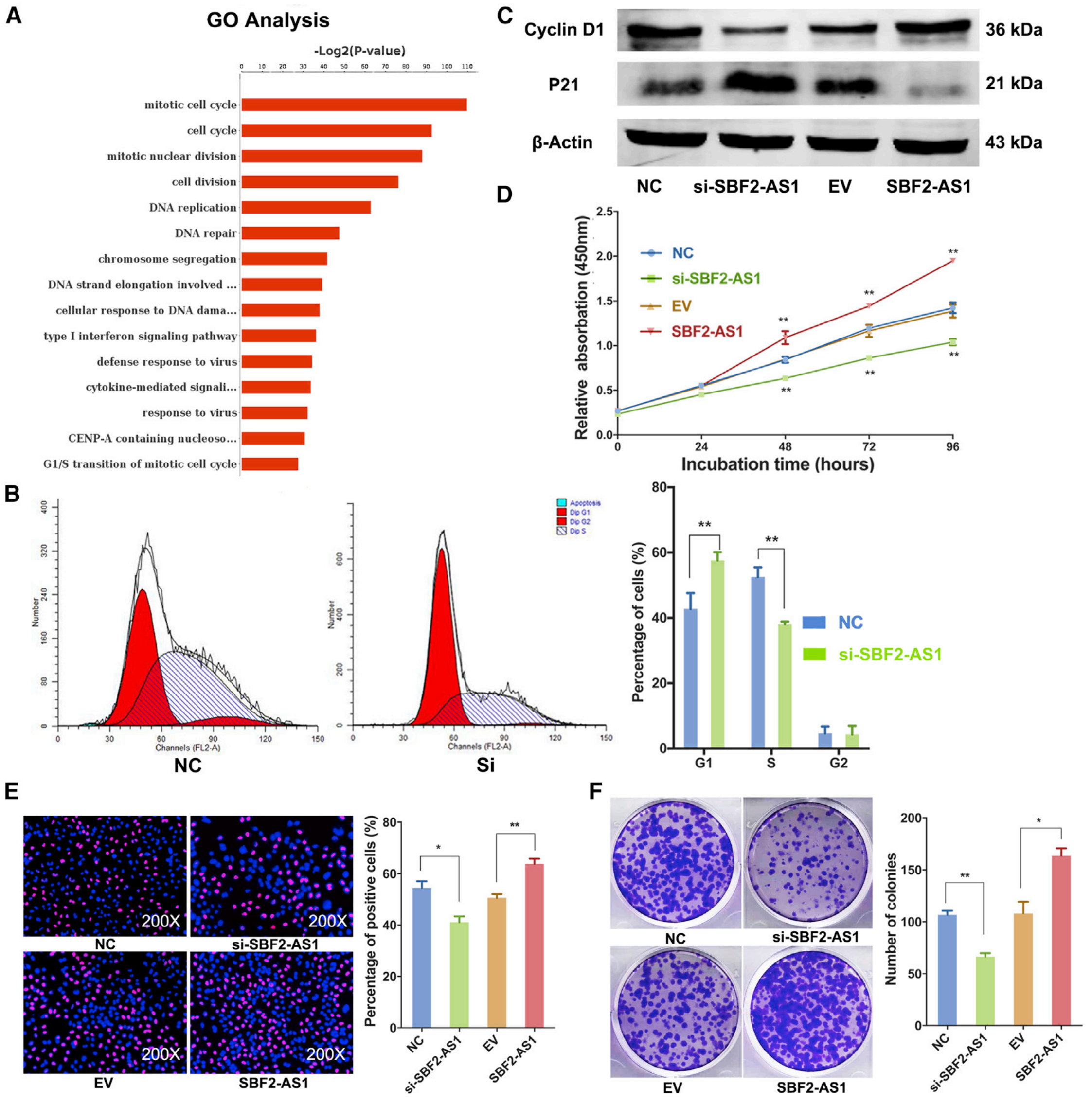

Figure 3. SBF2-AS1 Promotes Proliferation of LUAD Cells

The deregulated genes after the silence of SBF2-AS1 were significantly enriched in biological processes of the cell cycle (A). The cell cycle was arrested at G1 phase in A549 cells upon SBF2-AS1 knockdown (B). Cyclin D1 and P21 expression after silence and overexpression of SBF2-AS1 (C). Knockdown of SBF2-AS1 inhibited A549 cell proliferation but overexpression of SBF2-AS1 increased cell proliferation activity, as revealed by CCK-8 (D), EdU (E), and colony formation (F) assays. $^{*}p < 0.05$; $^{**}p < 0.01$. Error bars stand for mean and SE.

E2F1 Is a Downstream Target of miR-338-3p and miR-362-3p

Given that many cell-cycle-related genes were downregulated upon SBF2-AS1 knockdown (Table S2) and that SBF2-AS1 could bind with miRNA miR-338-3p and miR-362-3p, we therefore hypothesized that SBF2-AS1 may regulate cell-cycle-related genes through miR-338-3p and miR-362-3p. To this end, we retrieved 144 genes, which were downregulated after SBF2-AS1 knockdown and involved in items of cell cycles or proliferation according to Gene Ontology (GO) and Kyoto Encyclopedia of Genes and Genomes (KEGG) annotation (Table S5). Based on the number of GO items or pathways they involved, 19 predominant genes were identified (Table S5). A Venn plot was performed to identify ceRNA targets of SBF2-AS1 using the target genes of miR-338-3p and miR-362-3p and the 19 genes. As shown, 2 genes (CDC7 and CDC25A) were targets of miR-338-3p, 2 genes (PTTG1 and MAD2L1) were targets of miR-362-3p, and 4 genes (E2F1, CHEK1, CDC6, and CDK1) were targets of both miR-338-3p and miR-362-3p (Figure 4G). All 8 genes decreased after SBF2-AS1 knockdown, while E2F1 was most downregulated (Figure 4H); therefore, we selected E2F1 for further validation.

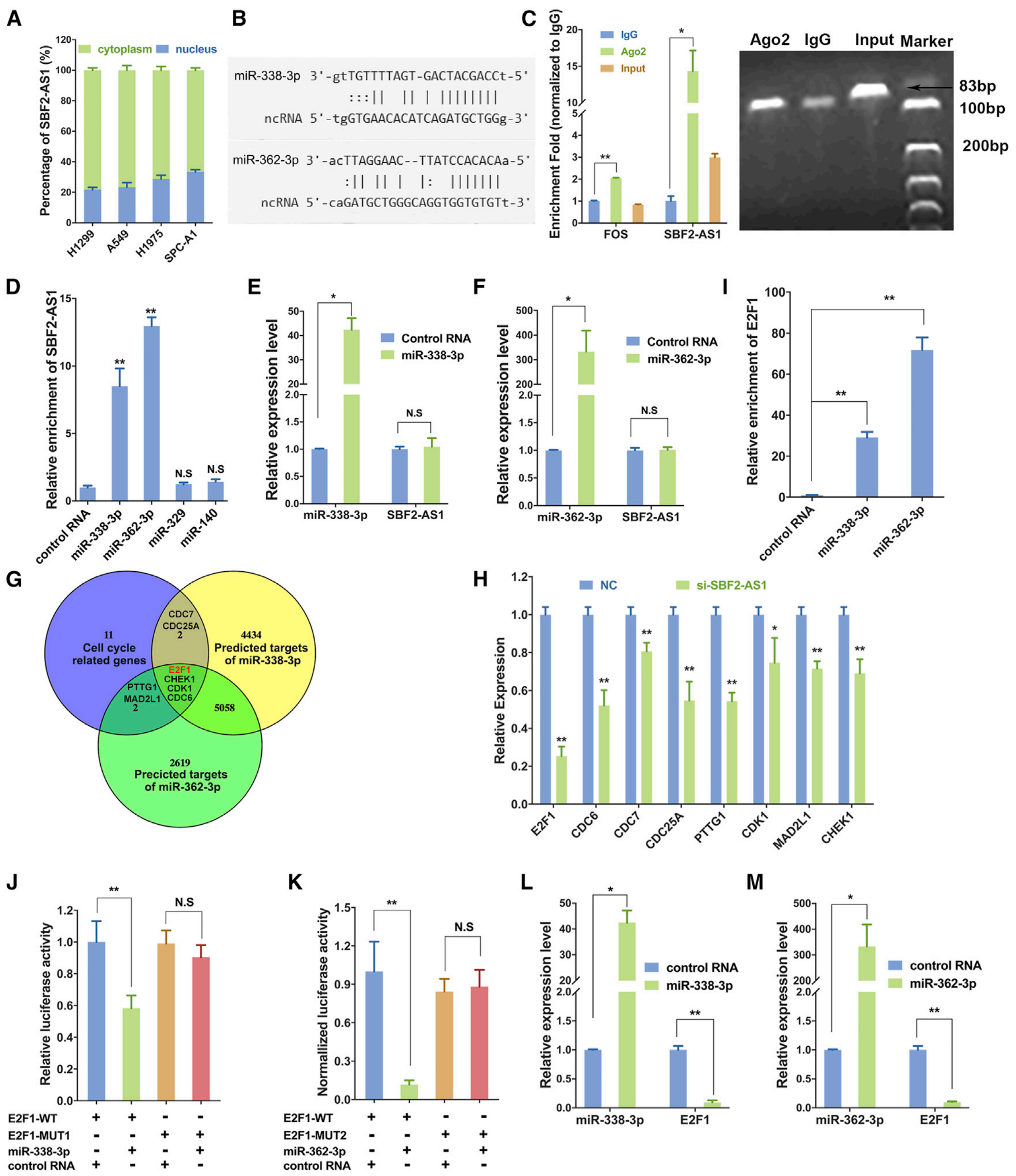

Figure 4. SBF2-AS1 Binds with miR-338-3p and miR-362-3p

SBF2-AS1 is located in both cytoplasm and nucleus in LUAD cells (A). PAR-CLIP-seq data suggested that SBF2-AS1 could bind with miR-338-3p and miR-362-3p (B). RIP assay using antibody specifically targeting Ago2 showed that SBF2-AS1 could bind with Ago2 protein in A549 cells (C). Biotin-coupled miRNA pull-down assay showed that SBF2-AS1 was significantly enriched by miR-338-3p and miR-362-3p (D). Compared with control RNA, overexpression of miR-338-3p (E) or miR-362-3p (F) did not alter SBF2-AS1 expression. E2F1 is the target of miR-338-3p and miR-362-3p. Venn plot showed that 8 cell-cycle-related genes were overlapped with predicted targets of miR-338-3p and miR-362-3p (G). Blue circle: cell-cycle-related genes that were selected from downregulated genes upon SBF2-AS1 knockdown; yellow circle: predicted targets of miR-338-3p; green circle: predicted targets of miR-362-3p. E2F1 was mostly downregulated upon SBF2-AS1 knockdown (H). Biotin-labeled miRNA pull-down assay showed that miR-338-3p and miR-362-3p could bind with E2F1 (I). In a dual-luciferase reporter assay, luciferase activity was inhibited by miR-338-3p (J) and miR-362-3p (K), but the inhibition was abolished when the binding sites of miR-338-3p (J) and miR-362-3p (K) were mutated, respectively. E2F1 mRNA expression level decreased upon ectopic expression of miR-338-3p (L) or miR-362-3p (M). *p < 0.05; **p < 0.01; N.S, no statistical significance. Error bars stand for mean and SE.

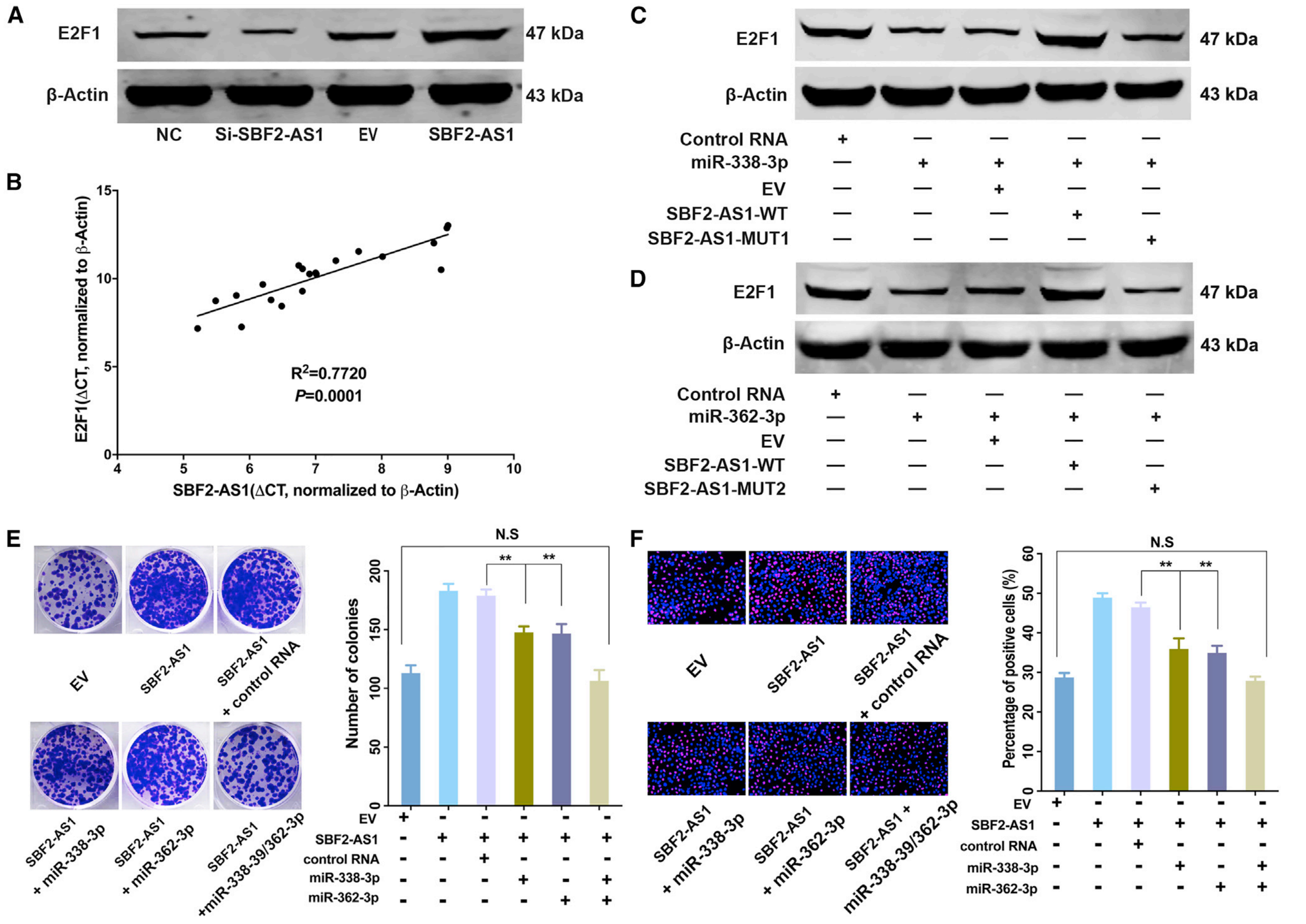

Figure 5. SBF2-AS1-miR-338-3p/362-3p-E2F1 Axis Promotes LUAD Tumorigenesis

E2F1 protein level decreased as SBF2-AS1 was knocked down and increased as SBF2-AS1 was overexpressed (A). Expression of SBF2-AS1 and expression of E2F1 were positively correlated in LUAD tissues ($p = 0.0001$, $R^2 = 0.772$) (B). E2F1 protein level was decreased by miR-338-3p and miR-362-3p and could be restored by SBF2-AS2, but after mutation of miR-338-3p (C) or miR-362-3p (D) binding sites, the SBF2-AS1-MUT could not restore E2F1 expression (C and D). Overexpression of SBF2-AS1 increased A549 cell proliferation activity, while the increase could be partially reversed by both miR-338-3p and miR-362-3p, and co-administration of miR-338-3p and miR-362-3p completely abolished the promoting effect driven by SBF2-AS1; colony formation assay was used in (E), and EdU assay was used in (F). *$p < 0.05$, **$p < 0.01$; N.S, no statistical significance. Error bars stand for mean and SE.

Biotin-labeled miRNA pull-down assay showed that miR-338-3p and miR-362-3p could bind with E2F1 (Figure 4I). A dual-luciferase reporter gene assay confirmed that miR-338-3p and miR-362-3p could bind to the 3′ UTR of E2F1 and significantly inhibit luciferase activity, whereas when the binding sites of miR-338-3p and miR-362-3p were deletion-mutated, the inhibition was reversed (Figures 4J and 4K). In addition, ectopic expression of both miR-338-3p and miR-362-3p could inhibit expression of E2F1 (Figures 4L and 4M). Together, these results revealed that E2F1 is target of miR-338-3p and miR-362-3p.

SBF2-AS1-miR-338-3p/362-3p-E2F1 Axis Promotes LUAD Tumorigenesis

As shown, E2F1 protein decreased when SBF2-AS1 was knocked down and increased when SBF2-AS1 was overexpressed (Figure 5A). In addition, expression of SBF2-AS1 and expression of E2F1 were positively correlated in lung cancer tissues (Figure 5B). We next sought to determine whether SBF2-AS1 regulates E2F1 expression by sponging miR-338-3p and miR-362-3p. To this end, we constructed 2 deletion-mutated SBF2-AS1 expression vectors, of which the binding sites of miR-338-3p (SBF2-AS1-MUT1) and miR-362-3p (SBF2-AS1-MUT2) were deletion-mutated, respectively (plasmid sequence is provided in Data S1). As shown, E2F1 expression was decreased by miR-338-3p, while ectopic expression of SBF2-AS1 restored E2F1 expression. However, when miR-338-3p binding sites were mutated, the mutated SBF2-AS1 failed to restore E2F1 expression (Figure 5C). The same results were also observed for miR-362-3p (Figure 5D).

Then, we proposed that SBF2-AS1 could promote lung cancer cell proliferation through the SBF2-AS1-miR-338-3p/362-3p-E2F1 axis.

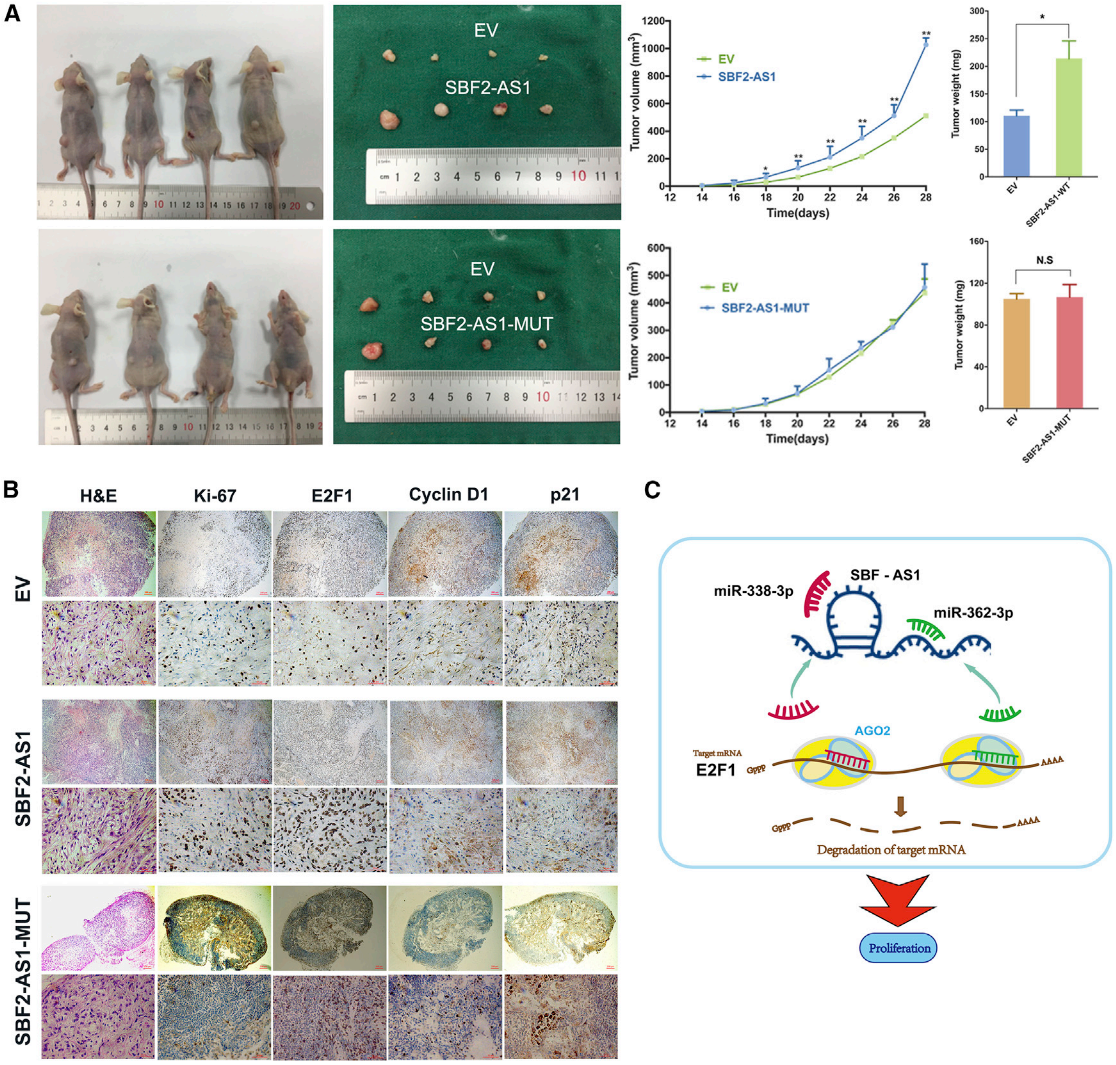

Figure 6. SBF2-AS1 Promotes LUAD Tumorigenesis *In Vivo*

SBF2-AS1 overexpression promoted lung cancer growth in a nude-mouse xenograft model; however, when the binding sites of miR-338-3p and miR-362-3p were both mutated, SBF2-AS1-MUT failed to promote growth of lung cancer (A). H&E staining and IHC staining of xenograft tumor tissues (B). The mechanic diagram of SBF2-AS1 promoting LUAD tumorigenesis (C). *p < 0.05, N.S., no statistical significance, and error bars stand for mean and SE.

A colony formation assay suggested that ectopic expression of SBF2-AS1 increased the number of colonies; then miR-338-3p or miR-362-3p alone could partially reverse the increase, and the combination of miR-338-3p and miR-362-3p completely reversed the increasing effect of SBF2-AS1 (Figure 5E; Figure S3A). EdU (Figure 5F; Figure S3B) and CCK-8 (Figures S3C and S3D) assays showed similar results. Collectively, these data showed that SBF2-AS1 increases E2F1 by binding with miR-338-3P and miR-362-3p, and the SBF2-AS1/miR-338-3p/362-3p/E2F1 axis promotes proliferation of LUAD.

SBF2-AS1 Is a Novel Therapeutic Target for LUAD

Given its function in cell proliferation, we next examined the potential roles of SBF2-AS1 *in vivo*. An *in vivo* xenograft model showed that SBF2-AS1 overexpression promoted lung cancer growth; however, when the binding sites of miR-338-3p and miR-362-3p were both mutated (SBF2-AS1-MUT), SBF2-AS1 failed to promote growth of lung cancer (Figure 6A). Further analyses of the tumor samples with immunohistochemical (IHC) suggested that E2F1 staining intensity decreased when miR-338-3p and miR-362-3p binding sites were both mutated, in comparison with wild-type SBF2-AS1 (Figure 6B). These data suggested that SBF2-AS1 could be a potential therapeutic target for lung cancer.

DISCUSSION

Tumorigenesis of LUAD is an extremely complex process. Abnormal cell proliferation represents a main feature at the early stage of

tumorigenesis. So far, there are few studies focusing on the tumor-stage-specific lncRNAs. WGCNA could transform gene expression data into co-expression module and correlate these modules with clinical characteristics,[25] which is well suited for integrating complementary datasets. We analyzed TCGA LUAD data with WGCNA and finally filtered out 5 tumor-stage-specific lncRNAs in LUAD (Figure 1); the other 4 lncRNAs also need further investigation besides SBF2-AS1. Although we previously reported that SBF2-AS1 was highly expressed in non-small cell lung cancer (NSCLC),[17] the present data indicate that SBF2-AS1 is more critical for the tumorigenesis of LUAD but not LUSC.

According to a ceRNA hypothesis, we filtered out 8 cell-cycle-related genes based on miR-338-3p and miR-362-3p target prediction and gene expression profile after SBF2-AS1 knockdown. In addition to E2F1, the other 7 genes are also critical regulators of cell cycle, cell proliferation, or proto-oncogenic genes, such as CHEK1, CDC6, CDC7, and CDK1. Based on ceRNA hypothesis, the expression of RNA transcripts that harbor the same miRNA binding sites should be parallel. In this study, we validate that SBF2-AS1 could increase E2F1 expression by competitively binding with miR-338-3p and miR-362-3p, and E2F1 expression is positively correlated with SBF2-AS1. TCGA expression data suggest that all 8 genes are positively correlated with SBF2-AS1 in LUAD (Table S6). Considering that these genes are also potential targets of miR-338-3p and miR-362-3p, it is reasonable to infer that SBF2-AS1 might increase expression of the 8 genes with a ceRNA mechanism.

Further, there might exist a ceRNA network that consists of SBF2-AS1, miR-338-3p/362-3p, and cell-cycle-related genes in LUAD. A Venn plot shows that more than half of the 144 cell-cycle-related genes (84 of 144) are potential targets of miR-338-3p and miR-362-3p (Figure S4A). Among the 84 genes, we filtered 25 genes that can bind with miR-338-3p and miR-362-3p according to PAR-CLIP and high-throughput sequencing (PAR-CLIP-seq) and draw the potential ceRNA network driven by SBF2-AS1 (Figure S4B). We noticed that a recent study also found that SBF2-AS1 could sponge miR-338-3p and promote angiogenesis in glioblastoma,[26] which is consistent with our data. More importantly, since there are many miRNA binding sites within the SBF2-AS1 sequence, our findings might be only a small part of the whole picture of the SBF2-AS1-driven ceRNA network.

E2F1 is a well-known transcription factor that promotes expression of various genes involved in cell cycle, cell proliferation, DNA repair, and other vital biological processes.[27] As a transcription factor, E2F1 could promote the expression of various lncRNAs.[28,29] In this study, we showed that lncRNA SBF2-AS1 could increase E2F1 expression, which adds new knowledge to the regulatory mechanism of E2F1 and provides insights into the complexity of cross-talk between lncRNAs and coding genes.

We demonstrate that SBF2-AS1 could promote cell proliferation and tumorigenesis of LUAD with *in vitro* and *in vivo* experiments. Besides lung cancer, TCGA data suggest that SBF2-AS1 is overexpressed in various kinds of cancer, including renal clear cell carcinoma, head and neck squamous cell carcinoma, and liver cancer. SBF2-AS1 may also play important roles in the tumorigenesis of these cancers. Intratumor injection of siRNA efficiently inhibited LUAD growth, suggesting that SBF2-AS1 could be a potential therapeutic target.

In summary, we find an early-stage, LUAD-specific lncRNA, SBF2-AS1, that is an unfavorable prognostic marker and a potential therapeutic target for LUAD. SBF2-AS1 functions as a sponge of miR-338-3p and miR-362-3p to increase the expression of E2F1 and promote LUAD tumorigenesis. Our findings demonstrate that one lncRNA contains various binding sites for different miRNAs, highlighting the complexity of the ceRNA regulatory network.

MATERIALS AND METHODS

Patients and Tissue Samples

All primary LUAD tissues and adjacent normal tissues were collected from patients who had undergone surgery at the Department of Thoracic Surgery, Jiangsu Cancer Hospital, 2012–2013. Experienced pathologists confirmed all tumors and paired adjacent tissues. Written informed consent was obtained from all patients. This study was approved by the Ethics Committee of Jiangsu Cancer Hospital and performed in accordance with the provisions of the Ethics Committee of Nanjing Medical University. All methods were performed in accordance with the relevant guidelines and regulations.

Cell Culture

A549, H1975, SPC-A1, and H1299 cells were purchased from the Shanghai Cell Bank of the Chinese Academy of Sciences. A549, H1975, and H1299 cells were cultured in RPMI 1640 medium; and SPC-A1 cells were cultured in DMEM medium supplemented with 10% fetal bovine serum (FBS) (GIBCO-BRL, Invitrogen, Carlsbad, CA, USA), 100 U/mL penicillin, and 100 mg/mL streptomycin in humidified air at 37°C with 5% CO_2. Authentication of A549, H1299, H1975, and SPC-A1 was verified by short tandem repeat DNA profiling within 6 months of use for the present study. The cells used in experiments were within 10 passages from thawing.

RNA Extraction and Real-Time qPCR Analysis

RNA was extracted from tissues or cultured cells with TRIzol Reagent according to the manufacturer's protocol (Life Technologies, Scotland, UK). RNA was reverse-transcribed with Prime Script RT Master Mix (Takara, catalog no. RR036A). Real-time qPCR was performed using SYBR Select Master Mix (Applied Biosystems, catalog no. 4472908) with the ABI7300 system (Applied Biosystems, Foster City, CA, USA) according to the manufacturer's instructions. All primers used are listed in Table S1. The ΔΔCt or ΔCt method was used to determine fold changes in subsequent calculations.

RNA Sequencing

A549 cells were plated in a 6-well plate and transfected with siRNA targeting SBF2-AS1 or negative control. 24 h after transfection, cells were harvested for RNA extraction and the subsequent library

construction and sequencing. RNA sequencing was performed by Novel Bioinformatics, and the raw data could be accessed in the GEO database (GEO: GSE103016).

RNA Isolation of Nuclear and Cytoplasmic Fractions

The subcellular localization of SBF2-AS1 was detected using the PARIS Kit according to the manufacturer's protocol (Ambion, Life Technologies, Carlsbad, CA, USA).

siRNA, Plasmid Construction, and Cell Transfection

siRNAs were provided by GenScript (Nanjing, China). miRNA mimics and primers were provided by RiboBio (Guangzhou, China). The full-length cDNA of human SBF2-AS1 was synthesized by Invitrogen (Shanghai, China) and cloned into the expression vector pCDNA3.1 (Clontech Laboratories, San Francisco, CA, USA). The final construct was verified by sequencing. Plasmid vectors for transfection were prepared using DNA Midiprep kits (E.Z.N.A. Endo-Free Plasmid Mini Kit II). The siRNAs and miRNA mimics were transfected using Lipofectamine iMAX (Invitrogen, Shanghai, China), and plasmids were transfected with X-tremeGENE (Roche Applied Science) according to the manufacturer's instructions. All siRNA sequences are listed in Table S1.

Cell Proliferation

Cells were harvested 24 h post-transfection, and the Cell Counting Kit-8 (CCK-8) assay was used to determine cell growth according to the manufacturer's instructions (Nanjing KeyGen Biotech., Nanjing, China). The EdU assay was performed by using a RiboBio kit (Guangzhou, China). After transfection, cells were stained with the BD Cycletest Plus DNA Reagent Kit (BD Biosciences) or the FITC Annexin V Apoptosis Detection Kit (BD Biosciences) according to the manufacturer's recommendations. Cells were then analyzed with a flow cytometer (BD FACScan; BD Biosciences) equipped with CellQuest software (BD Biosciences).

RIP

RIP experiments were performed using a Magna RIP RNA-Binding Protein Immunoprecipitation Kit (Millipore) according to the manufacturer's instructions. The A549 cells with a density of 80%–90% were collected in a cell culture dish with a diameter of 15 cm, and the number of the cells was about 3×10^7. Then the cells were lysed in RIP lysis buffer, and the cell extract was incubated with magnetic beads conjugated with anti-Ago2 or control anti-immunoglobulin G (IgG) antibody (Millipore) for 6 h at 4°C. The beads were washed and incubated with Proteinase K to remove proteins.

Biotin-Coupled miRNA Capture

The biotin-labeled miRNA pull-down assay was performed as previously described.[30] Briefly, the 3′−end biotinylated miRNA mimic or control biotin RNA (RiboBio) was transfected into A549 cells at a final concentration of 20 nM for 24 h. The biotin-coupled RNA complex was pulled down by incubating the cell lysates with streptavidin-coated magnetic beads (Life Technologies). The abundance of target RNAs in bound fractions was detected by real-time qPCR analysis.

Luciferase Reporter Assays

The E2F1 binding sites of miRNA were predicted by TargetScan (http://www.targetscan.org/vert_71/). The different fragment sequences were synthesized and then inserted into the pGL3-basic vector (Promega, Madison, WI, USA). All vectors were verified by sequencing, and luciferase activity was assessed using the Dual Luciferase Assay Kit (Promega) according to the manufacturer's instructions.

Animal Care and Xenograft Tumor Model

Female athymic BALB/c nude mice (4 weeks old) were maintained under specific pathogen-free conditions and manipulated according to protocols approved by the Nanjing Medical Experimental Animal Care Commission. In the tumor formation assay, for each mouse, 1×10^6 cells were subcutaneously injected in a single side, and both the right and left sides were injected with cells transfected with empty vector or constructed plasmids, respectively. Tumor growth was examined every week, and tumor volume was calculated using the following equation: $V = 0.5 \times D \times d^2$ (V, volume; D, longitudinal diameter; d, latitudinal diameter).

Western Blot Analysis

Cells were harvested, and protein was extracted from transfected cells and quantified as previously described[31] using 12% or 4%–20% polyacrylamide gradient SDS gel. Anti-β-actin and anti-SUZ12 were from Abcam (Hong Kong, China). Anti-p21, anti-Cyclin E1, anti-E2F1, and anti-Cyclin D1 were from Cell Signaling Technology (Boston, MA, USA).

WGCNA

The raw gene counts and fragments per kilobase of exon per million reads mapped (FPKM) data from TCGA lung adenocarcinoma project was downloaded from the data repository of TCGA with GDC-client (https://www.cancer.gov/about-nci/organization/ccg/research/structural-genomics/tcga). The combined dataset of GSE19804[18] contained a total of 120 early-stage LUAD samples hybridized to probesets present on the HG-U133A Plus2 platform (https://www.ncbi.nlm.nih.gov/geo/).

WGCNA was carried out on the differentially expressed genes using the R "WGCNA" package (v1.61). The weighted gene co-expression network was constructed using genes that were expressed at an FPKM value of 0.1 or higher in at least one of the samples. A soft power parameter was estimated and used to derive a correlation matrix for selected genes using the similarity measure, and the dynamic hybrid cut tree method was used to detect the sample clusters.[16,32]

Statistical Analysis

Differences between groups were assessed using a paired two-tailed Student's t test. One-way ANOVA or the nonparametric Kruskal-Wallis test was applied to assess the relationship between SBF2-AS1 expression and other characteristics. The strength of the association

between continuous variables was tested with the Spearman correlation. All statistical analyses were performed using SPSS 20 software (Abbott Laboratories, Chicago, IL, USA).

SUPPLEMENTAL INFORMATION

Supplemental Information can be found online at https://doi.org/10.1016/j.omtn.2019.04.004.

AUTHOR CONTRIBUTIONS

M.Q., R.Y., R.C., J.W., and L.X. designed the experiments. M.Q., R.C., W.X., S.W., Y.X., Z.M., W.X., J.W., T.F., Q.Z., and G.D. performed the experiments. M.Q., R.C., W.X., R.Y., and L.X. performed the data analysis. M.Q., R.C., R.Y., W.C.-S.C., P.C.M., G.B., S.T., P.U., G.M., and H.H.P. wrote the manuscript.

CONFLICTS OF INTERESTS

The authors declare no competing interests.

ACKNOWLEDGMENTS

The National Natural Science Foundation of China (81372321, 81472200, 81572261, 81702377, and 81702256), the Innovation Capability Development Project of Jiangsu Province (BM2015004), the Project of Jiangsu Provincial Medical Talent (ZDRCA2016033), the Natural Science Foundation of Beijing (7182169), and the Key Project of Cutting-edge Clinical Technology of Jiangsu Province (BE2016797) supported this work. M.Q. was supported in part by the Postdoctoral Fellowship of the Peking-Tsinghua Center for Life Sciences.

REFERENCES

1. Jemal, A., Siegel, R., Xu, J., and Ward, E. (2010). Cancer statistics, 2010. CA Cancer J. Clin. *60*, 277–300.
2. Jemal, A., Bray, F., Center, M.M., Ferlay, J., Ward, E., and Forman, D. (2011). Global cancer statistics. CA Cancer J. Clin. *61*, 69–90.
3. Hirsch, F.R., Scagliotti, G.V., Mulshine, J.L., Kwon, R., Curran, W.J., Jr., Wu, Y.L., and Paz-Ares, L. (2017). Lung cancer: current therapies and new targeted treatments. Lancet *389*, 299–311.
4. Lee, P.N., Forey, B.A., Coombs, K.J., Lipowicz, P.J., and Appleton, S. (2016). Time trends in never smokers in the relative frequency of the different histological types of lung cancer, in particular adenocarcinoma. Regul. Toxicol. Pharmacol. *74*, 12–22.
5. Zhang, L., Li, M., Wu, N., and Chen, Y. (2015). Time trends in epidemiologic characteristics and imaging features of lung adenocarcinoma: a population study of 21,113 cases in China. PLoS ONE *10*, e0136727.
6. Khurana, E., Fu, Y., Chakravarty, D., Demichelis, F., Rubin, M.A., and Gerstein, M. (2016). Role of non-coding sequence variants in cancer. Nat. Rev. Genet. *17*, 93–108.
7. Jinesh, G.G., Sambandam, V., Vijayaraghavan, S., Balaji, K., and Mukherjee, S. (2018). Molecular genetics and cellular events of K-Ras-driven tumorigenesis. Oncogene *37*, 839–846.
8. Zaratiegui, M., Irvine, D.V., and Martienssen, R.A. (2007). Noncoding RNAs and gene silencing. Cell *128*, 763–776.
9. Schmitt, A.M., and Chang, H.Y. (2016). Long noncoding RNAs in cancer pathways. Cancer Cell *29*, 452–463.
10. Qiu, M.T., Hu, J.W., Yin, R., and Xu, L. (2013). Long noncoding RNA: an emerging paradigm of cancer research. Tumour Biol. *34*, 613–620.
11. Engreitz, J.M., Ollikainen, N., and Guttman, M. (2016). Long non-coding RNAs: spatial amplifiers that control nuclear structure and gene expression. Nat. Rev. Mol. Cell Biol. *17*, 756–770.
12. Bhan, A., Soleimani, M., and Mandal, S.S. (2017). Long noncoding RNA and cancer: a new paradigm. Cancer Res. *77*, 3965–3981.
13. Lin, A., Hu, Q., Li, C., Xing, Z., Ma, G., Wang, C., Li, J., Ye, Y., Yao, J., Liang, K., et al. (2017). The LINK-A lncRNA interacts with PtdIns(3,4,5)P_3 to hyperactivate AKT and confer resistance to AKT inhibitors. Nat. Cell Biol. *19*, 238–251.
14. Zhou, C., Huang, C., Wang, J., Huang, H., Li, J., Xie, Q., Liu, Y., Zhu, J., Li, Y., Zhang, D., et al. (2017). LncRNA MEG3 downregulation mediated by DNMT3b contributes to nickel malignant transformation of human bronchial epithelial cells via modulating PHLPP1 transcription and HIF-1α translation. Oncogene *36*, 3878–3889.
15. Qiu, M., Xu, Y., Wang, J., Zhang, E., Sun, M., Zheng, Y., Li, M., Xia, W., Feng, D., Yin, R., and Xu, L. (2015). A novel lncRNA, LUADT1, promotes lung adenocarcinoma proliferation via the epigenetic suppression of p27. Cell Death Dis. *6*, e1858.
16. Langfelder, P., and Horvath, S. (2008). WGCNA: an R package for weighted correlation network analysis. BMC Bioinformatics *9*, 559.
17. Lv, J., Qiu, M., Xia, W., Liu, C., Xu, Y., Wang, J., Leng, X., Huang, S., Zhu, R., Zhao, M., et al. (2016). High expression of long non-coding RNA SBF2-AS1 promotes proliferation in non-small cell lung cancer. J. Exp. Clin. Cancer Res. *35*, 75.
18. Lu, T.P., Tsai, M.H., Lee, J.M., Hsu, C.P., Chen, P.C., Lin, C.W., Shih, J.Y., Yang, P.C., Hsiao, C.K., Lai, L.C., and Chuang, E.Y. (2010). Identification of a novel biomarker, SEMA5A, for non-small cell lung carcinoma in nonsmoking women. Cancer Epidemiol. Biomarkers Prev. *19*, 2590–2597.
19. Bild, A.H., Yao, G., Chang, J.T., Wang, Q., Potti, A., Chasse, D., Joshi, M.B., Harpole, D., Lancaster, J.M., Berchuck, A., et al. (2006). Oncogenic pathway signatures in human cancers as a guide to targeted therapies. Nature *439*, 353–357.
20. Győrffy, B., Surowiak, P., Budczies, J., and Lánczky, A. (2013). Online survival analysis software to assess the prognostic value of biomarkers using transcriptomic data in non-small-cell lung cancer. PLoS ONE *8*, e82241.
21. Salmena, L., Poliseno, L., Tay, Y., Kats, L., and Pandolfi, P.P. (2011). A ceRNA hypothesis: the Rosetta Stone of a hidden RNA language? Cell *146*, 353–358.
22. Thomson, D.W., and Dinger, M.E. (2016). Endogenous microRNA sponges: evidence and controversy. Nat. Rev. Genet. *17*, 272–283.
23. Xue, Y., Ouyang, K., Huang, J., Zhou, Y., Ouyang, H., Li, H., Wang, G., Wu, Q., Wei, C., Bi, Y., et al. (2013). Direct conversion of fibroblasts to neurons by reprogramming PTB-regulated microRNA circuits. Cell *152*, 82–96.
24. Li, J.H., Liu, S., Zhou, H., Qu, L.H., and Yang, J.H. (2014). starBase v2.0: decoding miRNA-ceRNA, miRNA-ncRNA and protein-RNA interaction networks from large-scale CLIP-Seq data. Nucleic Acids Res. *42*, D92–D97.
25. Miller, J.A., Cai, C., Langfelder, P., Geschwind, D.H., Kurian, S.M., Salomon, D.R., and Horvath, S. (2011). Strategies for aggregating gene expression data: the collapseRows R function. BMC Bioinformatics *12*, 322.
26. Yu, H., Zheng, J., Liu, X., Xue, Y., Shen, S., Zhao, L., Li, Z., and Liu, Y. (2017). Transcription factor NFAT5 promotes glioblastoma cell-driven angiogenesis via SBF2-AS1/miR-338-3p-mediated EGFL7 expression change. Front. Mol. Neurosci. *10*, 301.
27. Alonso, M.M., Alemany, R., Fueyo, J., and Gomez-Manzano, C. (2008). E2F1 in gliomas: a paradigm of oncogene addiction. Cancer Lett. *263*, 157–163.
28. Gasri-Plotnitsky, L., Ovadia, A., Shamalov, K., Nizri-Megnaji, T., Meir, S., Zurer, I., Cohen, C.J., and Ginsberg, D. (2017). A novel lncRNA, GASL1, inhibits cell proliferation and restricts E2F1 activity. Oncotarget *8*, 23775–23786.
29. Brodie, S., Lee, H.K., Jiang, W., Cazacu, S., Xiang, C., Poisson, L.M., Datta, I., Kalkanis, S., Ginsberg, D., and Brodie, C. (2017). The novel long non-coding RNA TALNEC2, regulates tumor cell growth and the stemness and radiation response of glioma stem cells. Oncotarget *8*, 31785–31801.
30. Zheng, Q., Bao, C., Guo, W., Li, S., Chen, J., Chen, B., Luo, Y., Lyu, D., Li, Y., Shi, G., et al. (2016). Circular RNA profiling reveals an abundant circHIPK3 that regulates cell growth by sponging multiple miRNAs. Nat. Commun. *7*, 11215.
31. Yang, X., Zhang, Z., Qiu, M., Hu, J., Fan, X., Wang, J., Xu, L., and Yin, R. (2013). Glypican-5 is a novel metastasis suppressor gene in non-small cell lung cancer. Cancer Lett. *341*, 265–273.
32. Zhang, B., and Horvath, S. (2005). A general framework for weighted gene co-expression network analysis. Stat. Appl. Genet. Mol. Biol. *4*, Article17.

OMTN, Volume *16*

Supplemental Information

Long Noncoding RNA SBF2-AS1 Is Critical for Tumorigenesis of Early-Stage Lung Adenocarcinoma

Rui Chen, Wenjia Xia, Siwei Wang, Youtao Xu, Zhifei Ma, Weizhang Xu, Erbao Zhang, Jie Wang, Tian Fang, Quan'an Zhang, Gaochao Dong, William Chi-shing Cho, Patrick C. Ma, Giovanni Brandi, Simona Tavolari, Peter Ujhazy, Giulio Metro, Helmut H. Popper, Rong Yin, Mantang Qiu, and Lin Xu

Supplementary Materials

Supplementary Table1. Primers and siRNA sequences

Supplementary Table2. Differentially expressed genes after knockdown of SBF2-AS1

Supplementary Table3. Function enrichment analyses of differentially expressed genes after knockdown of SBF2-AS1

Supplementary Table4. miRNA binding sites within SBF2-AS1 sequence predicted by miRanda algorithm and CLIP-seq

Supplementary Table5. Cell cycle-related genes that were downregulated after knockdown of SBF2-AS1

Supplementary Table6. Co-expression between 19 cell cycle-related genes and SBF2-AS1 among cancers

Supplementary File 1. Constructed plasmids vectors used in this study.

The following plasmids were generated by inserting the indicated sequences in the Pezx-FR02 dual- Luciferase reporter vector.

E2F1-WT, wild type E2F1 3'UTR

CAGGGCTTGGAGGGACCAGGGTTTCCAGAGATGCTCACCTTGTCTCTGCA
GCCCTGGAGCCCCCTGTCCCTGGCCGTCCTCCCAGCCTGTTTGGAAACATT
TAATTTATACCCCTCTCCTCTGTCTCCAGAAGCTTCTAGCTCTGGGGTCTGG
CTACCGCTAGGAGGCTGAGCAAGCCAGGAAGGGAAGGAGTCTGTGTGGTG
TGTATGTGCATGCAGCCTACACCCACACGTGTGTACCGGGGGTGAATGTGT
GTGAGCATGTGTGTGTGCATGTACCGGGGAATGAAGGTGAACATACACCTC
TGTGTGTGCACTGCAGACACGCCCCAGTGTGTCCACATGTGTGTGCATGAG
TCCATGTGTGCGCGTGGGGGGGCTCTAACTGCACTTTCGGCCCTTTTGCTC
TGGGGGTCCCACAAGGCCCAGGGCAGTGCCTGCTCCCAGAATCTGGTGCT
CTGACCAGGCCAGGTGGGGAGGCTTTGGCTGGCTGGGCGTGTAGGACGGT
GAGAGCACTTCTGTCTTAAAGGTTTTTCTGATTGAAGCTTTAATGGAGCGT
TATTTATTTATCGAGGCCTCTTTGGTGAGCCTGGGGAATCAGCAAAGGGGA
GGAGGGGTGTGGGGTTGATACCCCAACTCCCTCTACCCTTGAGCAAGGGC
AGGGGTCCCTGAGCTGTTCTTCTGCCCCATACTGAAGGAACTGAGGCCTGG
GTGATTTATTTATTGGGAAAGTGAGGGAGGGAGACAGACTGACTGACAGC
CATGGGTGGTCAGATGGTGGGGTGGGCCCTCTCCAGGGGGCCAGTTCAGG
GCCCCAGCTGCCCCCCAGGATGGATATGAGATGGGAGAGGTGAGTGGGGG
ACCTTCACTGATGTGGGCAGGAGGGGTGGTGAAGGCCTCCCCCAGCCCAG
ACCCTGTGGTCCCTCCTGCAGTGTCTGAAGCGCCTGCCTCCCCACTGCTCT
GCCCCACCCTCCAATCTGCACTTTGATTTGCTTCCTAACAGCTCTGTTCCCT
CCTGCTTTGGTTTTAATAAATATTTTGATGACGTT

E2F1-MUT1, binding site of miR-338-3p was deletion-mutated

CAGGGCTTGGAGGGACCAGGGTTTCCAGAGATGCTCACCTTGTCTCTGCA
GCCCTGGAGCCCCCTGTCCCTGGCCGTCCTCCCAGCCTGTTTGGAAACATT
TAATTTATACCCCTCTCCTCTGTCTCCAGAAGCTTCTAGCTCTGGGGTCTGG
CTACCGCTAGGAGGCTGAGCAAGCCAGGAAGGGAAGGAGTCTGTGTGGTG
TGTATGTGCATGCAGCCTACACCCACACGTGTGTACCGGGGGTGAATGTGT
GTGAGCATGTGTGTGTGCATGTACCGGGGAATGAAGGTGAACATACACCTC
TGTGTGTGCACTGCAGACACGCCCCAGTGTGTCCACATGTGTGTGCATGAG
TCCATGTGTGCGCGTGGGGGGGCTCTAACTGCACTTTCGGCCCTTTTGCTC
TGGGGGTCCCACAAGGCCCAGGGCAGTGCCTGCTCCCAGAATCTGGTGCT
CTGACCAGGCCAGGTGGGGAGGCTTTGGCTGGCTGGGCGTGTAGGACGGT
GAGAGCACTTCTGTCTTAAAGGTTTTTCTGATTGAAGCTTTAATGGAGCGT
TATTTATTTATCGAGGCCTCTTTGGTGAGCCTGGGGAATCAGCAAAGGGGA
GGAGGGGTGTGGGGTTGATACCCCAACTCCCTCTACCCTTGAGCAAGGGC

AGGGGTCCCTGAGCTGTTCTTCTGCCCCATACACTGAGGCCTGGGTGATTT
ATTTATTGGGAAAGTGAGGGAGGGAGACAGACTGACTGACAGCCATGGGT
GGTCAGATGGTGGGGTGGGCCCTCTCCAGGGGGCCAGTTCAGGGCCCCAG
CTGCCCCCCAGGATGGATATGAGATGGGAGAGGTGAGTGGGGGACCTTCA
CTGATGTGGGCAGGAGGGGTGGTGAAGGCCTCCCCCAGCCCAGACCCTGT
GGTCCCTCCTGCAGTGTCTGAAGCGCCTGCCTCCCCACTGCTCTGCCCCAC
CCTCCAATCTGCACTTTGATTTGCTTCCTAACAGCTCTGTTCCCTCCTGCTT
TGGTTTTAATAAATATTTTGATGACGTT

E2F1-MUT2, binding sites of miR-362-3p were deletion-mutated
CAGGGCTTGGAGGGACCAGGGTTTCCAGAGATGCTCACCTTGTCTCTGCA
GCCCTGGAGCCCCCTGTCCCTGGCCGTCCTCCCAGCCTGTTTGGAAACATT
TAATTTATACCCCTCTCCTCTGTCTCCAGAAGCTTCTAGCTCTGGGGTCTGG
CTACCGCTAGGAGGCTGAGCAAGCCAGGAAGGGAAGGAGTCTGTGTGTGT
GCATGCAGCCTACACCCACACCCGGGGGTGAATGTGTGTGAGCATGTGTGT
GTGCATGTACCGGGGAATGAAGGTGAACATACACCTCTGTGTGTGCACTGC
AGACACGCCCCAGTGTGTCCACATGTGTGTGCATGAGTCCATGTGTGCGCG
TGGGGGGGCTCTAACTGCACTTTCGGCCCTTTTGCTCTGGGGGTCCCACAA
GGCCCAGGGCAGTGCCTGCTCCCAGAATCTGGTGCTCTGACCAGGCCAGG
TGGGGAGGCTTTGGCTGGCTGGGCGTGTAGGACGGTGAGAGCACTTCTGT
CTTAAAGGTTTTTTCTGATTGAAGCTTTAATGGAGCGTTATTTATTTATCGAG
GCCTCTTTGGTGAGCCTGGGGAATCAGCAAAGGGGAGGAGGGGTGTGGG
GTTGATACCCCAACTCCCTCTACCCTTGAGCAAGGGCAGGGGTCCCTGAGC
TGTTCTTCTGCCCCATACTGAAGGAACTGAGGCCTGGGTGATTTATTTATTG
GGAAAGTGAGGGAGGGAGACAGACTGACTGACAGCCATGGGTGGTCAGA
TGGTGGGGTGGGCCCTCTCCAGGGGGCCAGTTCAGGGCCCCAGCTGCCCC
CCAGGATGGATATGAGATGGGAGAGGTGAGTGGGGGACCTTCACTGATGT
GGGCAGGAGGGGTGGTGAAGGCCTCCCCCAGCCCAGACCCTGTGGTCCCT
CCTGCAGTGTCTGAAGCGCCTGCCTCCCCACTGCTCTGCCCCACCCTCCAA
TCTGCACTTTGATTTGCTTCCTAACAGCTCTGTTCCCTCCTGCTTTGGTTTTA
ATAAATATTTTGATGACGTT

The following plasmids were generated by inserting the indicated sequences between the BamHI site and XhoI sites of pcDNA3.1(+) (Life Technologies).

SBF2-AS1 WT, wild SBF2-AS1 full length sequence
CAGGTTCCAGCCCCGACCCGGGCGCGCGGGGCCGACTAGGGTCGGGTCCA
GTGTGCGGTGGTCGCTCCGCTCCGGGCCGCTCCGCTCTGGGCGTCAGGGC
GCGGGGAGCTGCCCCGGGGTTCTGTCCACCGGGGAGGAAAGCCACGAGC
ACTGAGCGCCTCCTGAGAGCCAGCCCTGACGTGAACTCATTTATCTGCCA

CGACCCAGAAGGAGTCTACTGCTAAGATTTCAGCATGTCCTGTGGCTGAG
TTAATCAGAGTTATGACAGGAAGGTACCGGGCACACCATCGCAATGCTCC
ATCAATGCTAGTATGTTGTGTTCTTTCCTTCATATCAAGTCAACTCAAGCT
TGCTCTACTTACCTGGTGTACACAGTCTAAGAACTGTAAGAAGACTGGAG
CAAAACCACTCCCCTGACAGTTGAGGGTCAAGCTGCTCCTCTGACTGAAT
TTGTGACCAAAAGAGAGCCACTCTTTTTCAACCAACATCTGGAAGCCTTC
AAGTGTCCTATAAAAGGGATCACTGAGTAACTGAACCAGGGATGTCACCT
AGGGCATAAGCAGGATGGATTGTCATTAATTTTAGTTCTGAAAAAGGCCT
ATTACTAAGATAAAAGCACTTCCTTCTGATGATAGCTAATTCACAAATTTA
CCTGGACAGCAAATTTGTTCACTAACCATTCCAGGATGGCCAATAAAATT
AATTTTGTAAACTTGCCAGTAAAAACTAAAGCTCAAATTCATTTTGGCTAC
AAGTTTACTTTCAGGGAATTGAGACTTTAATCCTTAACTGGCCAGTTTGCT
GACAGCAACCTTTTCCTAAGTTAATCAGAGGCCAGAGGATCAGTTAAAAT
CAATTCTACTTTTTGGCAGATGCAGATTAATGTCTTAATGTAGTCTGAACT
CACACTTTTCACTCTGGAGTGAGTTAAGGCGGGGCTTATAGAGAGCCAGG
GGATGGACTGACAAAACCCAGGAAGGATGGTCCACGCCTCACACTGCTGG
TCAAGGGCTCCATCCGCAAGCCTGCATGGTACATCTGTGGATGGGAGAGC
TTGATGCCCGGAGCCAGAGCTCCAGGCTGCGTAGGGGCTAAGAGCTCATG
GTACGGAGTTTTCTGATGGATACCAGGTGCTGTTTAACACAGGACTTTCTC
TGTCCATAAACACATTAGTCATTATACAAAAGAGTCATCTGAAGAATTTA
AAAATGACCAAATGGAAATATAAATATAAAATATCAAAAAATATGTCTAA
TAGTTTACTTGTGCAGTGATGTCCCAGCCTTCCTCCAAACAGACCAAAACT
GAGGAACCATTCTCAAGTACTTCTGATACAACCACAGCCAGCTGCATTAT
CCTGTGAAGCTAAGAGACAGGAAGGAGAACACAATTAGACCAAGCGCTT
TCTGAGTGCAGAAGTCAGAGAGAGTTGCTTTCAACCCTGGATAATCAAAC
GACTTAGGGATAAAGCGCTTGCACAAAAGAACCACATAATGTTTTAAATA
CCTAAAGTTCTTTCAAATCAAGATTAAACAATGACACTTTTGACTGGATGC
AATGACTCATGCCCATAATCCCAGCACTTTGGGAGGCCGAGCCGGGCAGG
TCATTTGAGGTTCGGAGTTCGAGACCAGCCTGGGCAACATAAGCAAGACC
CTGTCTCGAAAAATAAAAATAAAAAAGATACTCCTATTTGTCTATACTGT
GTACTAAATGAGAAAAAAACTTTCAATATATTTGTGCTAAGTTTTTAAAGC
CACATCAATTGGCTCAAAGTCCTTAATAAACAATTAAGGTATGAGAATAG
TATATAGATTTTAAAACCAGATGTATAACATTGTAATTTTAAAAAATATTT
TAATGATCATCAGACATAATTTGCAATTTGAATCCAACTCTGAACAAGAA
CAAAAATGAAAAAACCCATCTCTTAAACAGAGCTAGGCTACTTTAAACAC
AGGAATAAGGCAAATGCCCTACTCAGTAATGAGAAGCTCCTCATGTAACT
GTTTCTACAACATAGCTATCACCAGGCCTGAATTCTTTTGGGCTCAGAATT
TGTTCTAAGAAATGGCTATTATTAAAAAGTCAAAAAATAACAGATGCTGG
CGAGCCTGTGGAGAAAAGGGAATGTATATACATTTCTGGTGGGAATGTAA
ATTAGTTCAGCCATTGTAGAAAGTAGTTTGGTGATTTCTCAAATAACTTAA
AACAGAATTACCATTTGACCTAGCAATCCCATTACTGAGTATATACCCAA
AGGAATATAAATTGTTCTACCATAAAGTCACATGCACACACATGTTCAGT
GCTGTACTATTCACAATAGCAAAGACATGGAGTCAACCTATATGCCCATC
AACGGTAGACTGGATAAAGAAAATATGGTACATATACACCATGGAATACT

ACACAGCCATAAAAAGAATGAGCTTATGTCCTTTGCAGTAACACAGCTGA
AGCAAACTAACACAGGAATGGAAAACCAAATACTGCATATTCTCACTTAT
AAGTGGGAGCTAAACATTGAGTACACATGGACACAAAGAAAGGAATAGA
CGCCTGGGGCGTATTTGAGGTTGGAGGTGAGAGGAGGGTGAGGATAGAA
AAACTACCTATCAGGTACTATGCTTATTACCTGGGTGATGAAATAATCTGT
ACACCAAACTCCCAAGATATGCAATGTACCTATACAACAAACCTACAT

SBF2-AS1-MUT1, **binding sites of miR-338-3p were deletion-mutated**
CAGGTTCCAGCCCCGACCCGGGCGCGGGGCCGACTAGGGTCGGGTCCA
GTGTGCGGTGGTCGCTCCGCTCCGGGCCGCTCCGCTCTGGGCGTCAGGGC
GCGGGGAGCTGCCCCGGGGTTCTGTCCACCGGGGAGGAAAGCCACGAGC
ACTGAGCGCCTCCTGAGAGCCAGCCCTGACGTGAACTCATTTTATCTGCCA
CGACCCAGAAGGAGTCTACTGCTAAGATTTCAGCATGTCCTGTGGCTGAG
TTAATCAGAGTTATGACAGGAAGGTACCGGGCACACCATCGCAATGCTCC
ATCAATGCTAGTATGTTGTGTTCTTTCCTTCATATCAAGTCAACTCAAGCT
TGCTCTACTTACCTGGTGTACACAGTCTAAGAACTGTAAGAAGACTGGAG
CAAAACCACTCCCCTGACAGTTGAGGGTCAAGCTGCTCCTCTGACTGAAT
TTGTGACCAAAAGAGAGCCACTCTTTTTCAACCAACATCTGGAAGCCTTC
AAGTGTCCTATAAAAGGGATCACTGAGTAACTGAACCAGGGATGTCACCT
AGGGCATAAGCAGGATGGATTGTCATTAATTTTAGTTCTGAAAAAGGCCT
ATTACTAAGATAAAAGCACTTCCTTCTGATGATAGCTAATTCACAAATTTA
CCTGGACAGCAAATTTGTTCACTAACCATTCCAGGATGGCCAATAAAATT
AATTTTGTAAACTTGCCAGTAAAAACTAAAGCTCAAATTCATTTTGGCTAC
AAGTTTACTTTCAGGGAATTGAGACTTTAATCCTTAACTGGCCAGTTTGCT
GACAGCAACCTTTTCCTAAGTTAATCAGAGGCCAGAGGATCAGTTAAAAT
CAATTCTACTTTTTGGCAGATGCAGATTAATGTCTTAATGTAGTCTGAACT
CACACTTTTCACTCTGGAGTGAGTTAAGGCGGGGCTTATAGAGAGCCAGG
GGATGGACTGACAAAACCCAGGAAGGATGGTCCACGCCTCACACTCAAG
GGCTCCATCCGCAAGCCTGCATGGTACATCTGTGGATGGGAGAGCTTGAT
GCCCGGAGCCAGAGCTCCAGGCTGCGTAGGGGCTAAGAGCTCATGGTACG
GAGTTTTCTGATGGATACCAGGTGCTGTTTAACACAGGACTTTCTCTGTCC
ATAAACACATTAGTCATTATACAAAAGAGTCATCTGAAGAATTTAAAAAT
GACCAAATGGAAATATAAATATAAAATATCAAAAAATATGTCTAATAGTT
TACTTGTGCAGTGATGTCCCAGCCTTCCTCCAAACAGACCAAAACTGAGG
AACCATTCTCAAGTACTTCTGATACAACCACAGCCAGCTGCATTATCCTGT
GAAGCTAAGAGACAGGAAGGAGAACACAATTAGACCAAGCGCTTTCTGA
GTGCAGAAGTCAGAGAGAGTTGCTTTCAACCCTGGATAATCAAACGACTT
AGGGATAAAGCGCTTGCACAAAAGAACCACATAATGTTTTAAATACCTAA
AGTTCTTTCAAATCAAGATTAAACAATGACACTTTTGACTGGATGCAATG
ACTCATGCCCATAATCCCAGCACTTTGGGAGGCCGAGCCGGGCAGGTCAT
TTGAGGTTCGGAGTTCGAGACCAGCCTGGGCAACATAAGCAAGACCCTGT
CTCGAAAAATAAAAATAAAAAAGATACTCCTATTTGTCTATACTGTGTAC
TAAATGAGAAAAAAACTTTCAATATATTTGTGCTAAGTTTTAAAGCCAC

ATCAATTGGCTCAAAGTCCTTAATAAACAATTAAGGTATGAGAATAGTAT
ATAGATTTTAAAACCAGATGTATAACATTGTAATTTTAAAAAATATTTTAA
TGATCATCAGACATAATTTGCAATTTGAATCCAACTCTGAACAAGAACAA
AAATGAAAAAACCCATCTCTTAAACAGAGCTAGGCTACTTTAAACACAGG
AATAAGGCAAATGCCCTACTCAGTAATGAGAAGCTCCTCATGTAACTGTT
TCTACAACATAGCTATCACCAGGCCTGAATTCTTTTGGGCTCAGAATTTGT
TCTAAGAAATGGCTATTATTAAAAAGTCAAAAAATAACACGAGCCTGTGG
AGAAAAGGGAATGTATATACATTTCTGGTGGGAATGTAAATTAGTTCAGC
CATTGTAGAAAGTAGTTTGGTGATTTCTCAAATAACTTAAAACAGAATTA
CCATTTGACCTAGCAATCCCATTACTGAGTATATACCCAAAGGAATATAA
ATTGTTCTACCATAAAGTCACATGCACACACATGTTCAGTGCTGTACTATT
CACAATAGCAAAGACATGGAGTCAACCTATATGCCCATCAACGGTAGACT
GGATAAAGAAAATATGGTACATATACACCATGGAATACTACACAGCCATA
AAAAGAATGAGCTTATGTCCTTTGCAGTAACACAGCTGAAGCAAACTAAC
ACAGGAATGGAAAACCAAATACTGCATATTCTCACTTATAAGTGGGAGCT
AAACATTGAGTACACATGGACACAAAGAAAGGAATAGACGCCTGGGGCG
TATTTGAGGTTGGAGGTGAGAGGAGGGTGAGGATAGAAAAACTACCTATC
AGGTACTATGCTTATTACCTGGGTGATGAAATAATCTGTACACCAAACTCC
CAAGATATGCAATGTACCTATACAACAAACCTACAT

SBF2-AS1-MUT2, **binding site of miR-362-3p was deletion-mutated**
CAGGTTCCAGCCCCGACCCGGGCGCGCGGGGCCGACTAGGGTCGGGTCCA
GTGTGCGGTGGTCGCTCCGCTCCGGGCCGCTCCGCTCTGGGCGTCAGGGC
GCGGGGAGCTGCCCCGGGGTTCTGTCCACCGGGGAGGAAAGCCACGAGC
ACTGAGCGCCTCCTGAGAGCCAGCCCTGACGTGAACTCATTTTATCTGCCA
CGACCCAGAAGGAGTCTACTGCTAAGATTTCAGCATGTCCTGTGGCTGAG
TTAATCAGAGTTATGACAGGAAGGTACCGGGCACACCATCGCAATGCTCC
ATCAATGCTAGTATGTTGTGTTCTTTCCTTCATATCAAGTCAACTCAAGCT
TGCTCTACTTACCTGGTGTACACAGTCTAAGAACTGTAAGAAGACTGGAG
CAAAACCACTCCCCTGACAGTTGAGGGTCAAGCTGCTCCTCTGACTGAAT
TTGTGACCAAAAGAGAGCCACTCTTTTTCAACCAACATCTGGAAGCCTTC
AAGTGTCCTATAAAAGGGATCACTGAGTAACTGAACCAGGGATGTCACCT
AGGGCATAAGCAGGATGGATTGTCATTAATTTTAGTTCTGAAAAAGGCCT
ATTACTAAGATAAAAGCACTTCCTTCTGATGATAGCTAATTCACAAATTTA
CCTGGACAGCAAATTTGTTCACTAACCATTCCAGGATGGCCAATAAAATT
AATTTTGTAAACTTGCCAGTAAAAACTAAAGCTCAAATTCATTTTGGCTAC
AAGTTTACTTTCAGGGAATTGAGACTTTAATCCTTAACTGGCCAGTTTGCT
GACAGCAACCTTTTCCTAAGTTAATCAGAGGCCAGAGGATCAGTTAAAAT
CAATTCTACTTTTTGGCAGATGCAGATTAATGTCTTAATGTAGTCTGAACT
CACACTTTTCACTCTGGAGTGAGTTAAGGCGGGGCTTATAGAGAGCCAGG
GGATGGACTGACAAAACCCAGGAAGGATGGTCCACGCCTCACACTGCTGG
TCAAGGGCTCCATCCGCAAGCCTGCATGGTACATCTGTGGATGGGAGAGC
TTGATGCCCGGAGCCAGAGCTCCAGGCTGCGTAGGGGCTAAGAGCTCATG

GTACGGAGTTTTCTGATGGATACCAGGTGCTGTTTAACACAGGACTTTCTC
TGTCCATAAACACATTAGTCATTATACAAAAGAGTCATCTGAAGAATTTA
AAAATGACCAAATGGAAATATAAATATAAAATATCAAAAAATATGTCTAA
TAGTTTACTTGTGCAGTGATGTCCCAGCCTTCCTCCAAACAGACCAAAACT
GAGGAACCATTCTCAAGTACTTCTGATACAACCACAGCCAGCTGCATTAT
CCTGTGAAGCTAAGAGACAGGAAGGAGAACACAATTAGACCAAGCGCTT
TCTGAGTGCAGAAGTCAGAGAGAGTTGCTTTCAACCCTGGATAATCAAAC
GACTTAGGGATAAAGCGCTTGCACAAAAGAACCACATAATGTTTTAAATA
CCTAAAGTTCTTTCAAATCAAGATTAAACAATGACACTTTTGACTGGATGC
AATGACTCATGCCCATAATCCCAGCACTTTGGGAGGCCGAGCCGGGCAGG
TCATTTGAGGTTCGGAGTTCGAGACCAGCCTGGGCAACATAAGCAAGACC
CTGTCTCGAAAAATAAAAATAAAAAAGATACTCCTATTTGTCTATACCTA
AATGAGAAAAAAACTTTCAATATATTTGTGCTAAGTTTTTAAAGCCACATC
AATTGGCTCAAAGTCCTTAATAAACAATTAAGGTATGAGAATAGTATATA
GATTTTAAAACCAGATGTATAACATTGTAATTTTAAAAAATATTTTAATGA
TCATCAGACATAATTTGCAATTTGAATCCAACTCTGAACAAGAACAAAAA
TGAAAAAACCCATCTCTTAAACAGAGCTAGGCTACTTTAAACACAGGAAT
AAGGCAAATGCCCTACTCAGTAATGAGAAGCTCCTCATGTAACTGTTTCT
ACAACATAGCTATCACCAGGCCTGAATTCTTTTGGGCTCAGAATTTGTTCT
AAGAAATGGCTATTATTAAAAAGTCAAAAAATAACAGATGCTGGCGAGC
CTGTGGAGAAAAGGGAATGTATATACATTTCTGGTGGGAATGTAAATTAG
TTCAGCCATTGTAGAAAGTAGTTTGGTGATTTCTCAAATAACTTAAAACAG
AATTACCATTTGACCTAGCAATCCCATTACTGAGTATATACCCAAAGGAA
TATAAATTGTTCTACCATAAAGTCACATGCACACACATGTTCAGTGCTGTA
CTATTCACAATAGCAAAGACATGGAGTCAACCTATATGCCCATCAACGGT
AGACTGGATAAAGAAAATATGGTACATATACACCATGGAATACTACACAG
CCATAAAAAGAATGAGCTTATGTCCTTTGCAGTAACACAGCTGAAGCAAA
CTAACACAGGAATGGAAAACCAAATACTGCATATTCTCACTTATAAGTGG
GAGCTAAACATTGAGTACACATGGACACAAAGAAAGGAATAGACGCCTG
GGGCGTATTTGAGGTTGGAGGTGAGAGGAGGGTGAGGATAGAAAAACTA
CCTATCAGGTACTATGCTTATTACCTGGGTGATGAAATAATCTGTACACCA
AACTCCCAAGATATGCAATGTACCTATACAACAAACCTACAT

SBF2-AS1-MUT, **binding sites of miR-338-3p and miR-362-3p were deletion-mutated**

CAGGTTCCAGCCCCGACCCGGGCGCGGGGCCGACTAGGGTCGGGTCCA
GTGTGCGGTGGTCGCTCCGCTCCGGGCCGCTCCGCTCTGGGCGTCAGGGC
GCGGGGAGCTGCCCCGGGGTTCTGTCCACCGGGGAGGAAAGCCACGAGC
ACTGAGCGCCTCCTGAGAGCCAGCCCTGACGTGAACTCATTTTATCTGCCA
CGACCCAGAAGGAGTCTACTGCTAAGATTTCAGCATGTCCTGTGGCTGAGT

TAATCAGAGTTATGACAGGAAGGTACCGGGCACACCATCGCAATGCTCCAT
CAATGCTAGTATGTTGTGTTCTTTCCTTCATATCAAGTCAACTCAAGCTTGC
TCTACTTACCTGGTGTACACAGTCTAAGAACTGTAAGAAGACTGGAGCAAA
ACCACTCCCCTGACAGTTGAGGGTCAAGCTGCTCCTCTGACTGAATTTGTG
ACCAAAAGAGAGCCACTCTTTTTCAACCAACATCTGGAAGCCTTCAAGTG
TCCTATAAAAGGGATCACTGAGTAACTGAACCAGGGATGTCACCTAGGGCA
TAAGCAGGATGGATTGTCATTAATTTTAGTTCTGAAAAAGGCCTATTACTAA
GATAAAAGCACTTCCTTCTGATGATAGCTAATTCACAAATTTACCTGGACAG
CAAATTTGTTCACTAACCATTCCAGGATGGCCAATAAAATTAATTTTGTAAA
CTTGCCAGTAAAAACTAAAGCTCAAATTCATTTTGGCTACAAGTTTACTTTC
AGGGAATTGAGACTTTAATCCTTAACTGGCCAGTTTGCTGACAGCAACCTT
TTCCTAAGTTAATCAGAGGCCAGAGGATCAGTTAAAATCAATTCTACTTTTT
GGCAGATGCAGATTAATGTCTTAATGTAGTCTGAACTCACACTTTTCACTCT
GGAGTGAGTTAAGGCGGGGCTTATAGAGAGCCAGGGGATGGACTGACAAA
ACCCAGGAAGGATGGTCCACGCCTCACACTCAAGGGCTCCATCCGCAAGC
CTGCATGGTACATCTGTGGATGGGAGAGCTTGATGCCCGGAGCCAGAGCTC
CAGGCTGCGTAGGGGCTAAGAGCTCATGGTACGGAGTTTTCTGATGGATAC
CAGGTGCTGTTTAACACAGGACTTTCTCTGTCCATAAACACATTAGTCATTA
TACAAAAGAGTCATCTGAAGAATTTAAAAATGACCAAATGGAAATATAAAT
ATAAAATATCAAAAAATATGTCTAATAGTTTACTTGTGCAGTGATGTCCCAG
CCTTCCTCCAAACAGACCAAAACTGAGGAACCATTCTCAAGTACTTCTGAT
ACAACCACAGCCAGCTGCATTATCCTGTGAAGCTAAGAGACAGGAAGGAG
AACACAATTAGACCAAGCGCTTTCTGAGTGCAGAAGTCAGAGAGAGTTGC
TTTCAACCCTGGATAATCAAACGACTTAGGGATAAAGCGCTTGCACAAAAG
AACCACATAATGTTTTAAATACCTAAAGTTCTTTCAAATCAAGATTAAACAA
TGACACTTTTGACTGGATGCAATGACTCATGCCCATAATCCCAGCACTTTGG
GAGGCCGAGCCGGGCAGGTCATTTGAGGTTCGGAGTTCGAGACCAGCCTG
GGCAACATAAGCAAGACCCTGTCTCGAAAAATAAAAATAAAAAAGATACT
CCTATTTGTCTATACCTAAATGAGAAAAAAACTTTCAATATATTTGTGCTAAG
TTTTTAAAGCCACATCAATTGGCTCAAAGTCCTTAATAAACAATTAAGGTAT
GAGAATAGTATATAGATTTTAAAACCAGATGTATAACATTGTAATTTTAAAAA
ATATTTTAATGATCATCAGACATAATTTGCAATTTGAATCCAACTCTGAACAA
GAACAAAAATGAAAAAACCCATCTCTTAAACAGAGCTAGGCTACTTTAAAC
ACAGGAATAAGGCAAATGCCCTACTCAGTAATGAGAAGCTCCTCATGTAAC
TGTTTCTACAACATAGCTATCACCAGGCCTGAATTCTTTTGGGCTCAGAATT
TGTTCTAAGAAATGGCTATTATTAAAAAGTCAAAAAATAACACGAGCCTGT
GGAGAAAAGGGAATGTATATACATTTCTGGTGGGAATGTAAATTAGTTCAG
CCATTGTAGAAAGTAGTTTGGTGATTTCTCAAATAACTTAAAACAGAATTAC
CATTTGACCTAGCAATCCCATTACTGAGTATATACCCAAAGGAATATAAATTG
TTCTACCATAAAGTCACATGCACACACATGTTCAGTGCTGTACTATTCACAA
TAGCAAAGACATGGAGTCAACCTATATGCCCATCAACGGTAGACTGGATAA
AGAAAATATGGTACATATACACCATGGAATACTACACAGCCATAAAAAGAAT
GAGCTTATGTCCTTTGCAGTAACACAGCTGAAGCAAACTAACACAGGAATG
GAAAACCAAATACTGCATATTCTCACTTATAAGTGGGAGCTAAACATTGAGT

ACACATGGACACAAAGAAAGGAATAGACGCCTGGGGCGTATTTGAGGTTG
GAGGTGAGAGGAGGGTGAGGATAGAAAAACTACCTATCAGGTACTATGCTT
ATTACCTGGGTGATGAAATAATCTGTACACCAAACTCCCAAGATATGCAATG
TACCTATACAACAAACCTACAT

Figure S1: Screening early stage-specific lncRNAs. 5 outliers were excluded from hierarchical clustering of cancer tissue samples (A). 12 significant co-expression gene modules across all 508 sampling sets were detected with WGCNAs (B). Soft thresholding power to achieve scale-free topology ($\beta = 2$) (C). The correlation within gene sets of module greenyellow (D). The heatmap of all 3250 differentially expressed genes in GSE19804 (E).

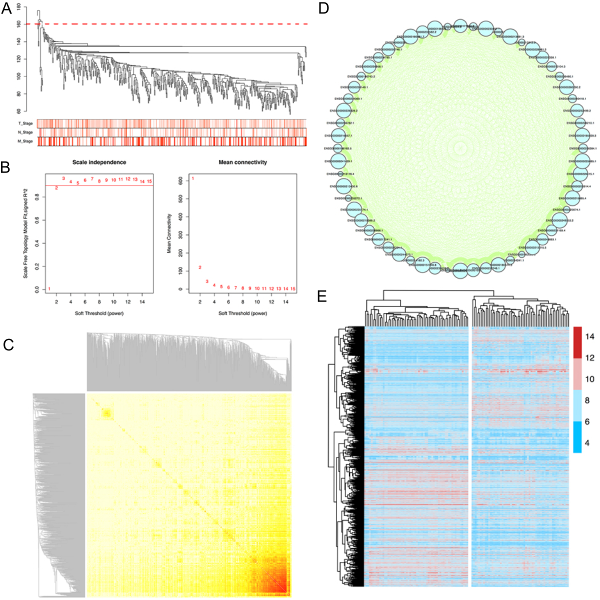
A
T_Stage
N_Stage
M_Stage
B
Scale independence
Mean connectivity
Scale Free Topology Model Fit,signed R^2
Mean Connectivity
Soft Threshold (power)
Soft Threshold (power)
C
D
E
14
12
10
8
6
4

Figure S2. SBF2-AS1promotes H1299 cell proliferation. Cell cycle was arrested at G1 phase in H1299 cells upon SBF2-AS1 knockdown (A). Expression of Cyclin D1 and P21 after ectopic expression and silence of SBF2-AS1 in H1299 cells (B). CCK8 assay (C), EdU (D), and colony formation assay (E) in H1299 cells after ectopic and silence of SBF2-AS1

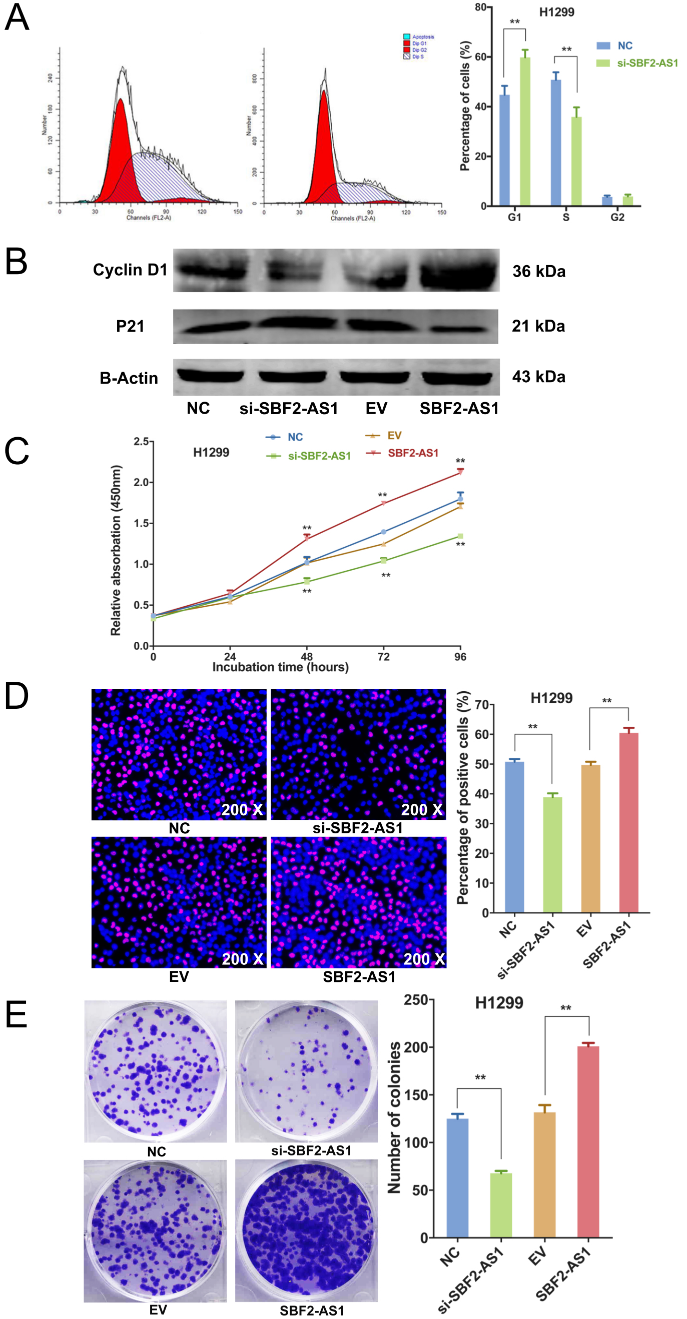
A
Apoptosis
Dip G1
Dip G2
Dip S
Number
Channels (FL2-A)
H1299
NC
si-SBF2-AS1
Percentage of cells (%)
G1
S
G2
B
Cyclin D1
36 kDa
P21
21 kDa
B-Actin
43 kDa
NC
si-SBF2-AS1
EV
SBF2-AS1
C
H1299
NC
si-SBF2-AS1
EV
SBF2-AS1
Relative absorbation (450nm)
Incubation time (hours)
D
200 X
NC
si-SBF2-AS1
EV
SBF2-AS1
H1299
Percentage of positive cells (%)
E
NC
si-SBF2-AS1
EV
SBF2-AS1
H1299
Number of colonies

Figure S3. Colony formation (A) and EdU (B) assay in H1299 cells. CCK8 assay in A549 (C) and H1299 (D) cells.

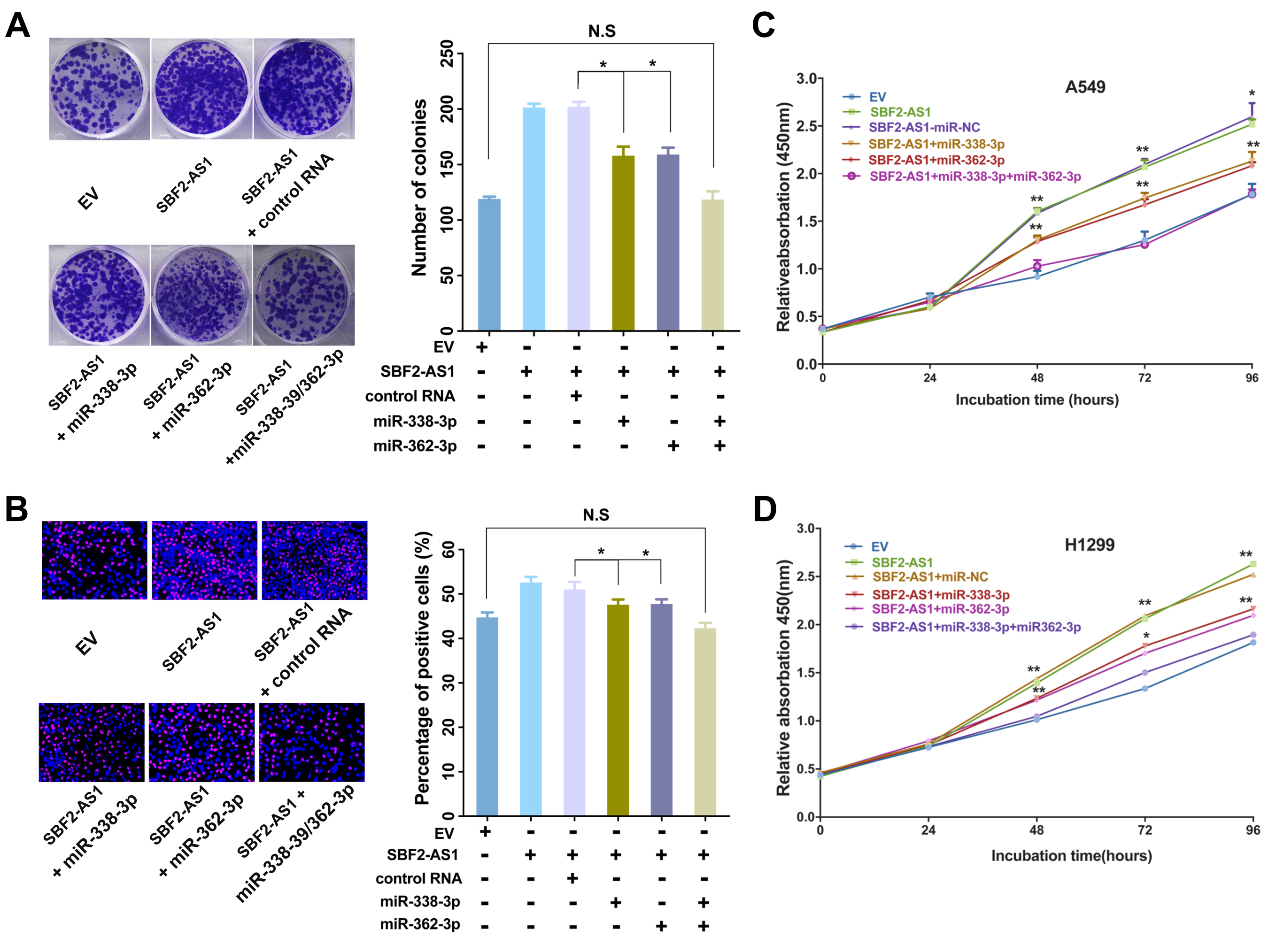
A
EV
SBF2-AS1
SBF2-AS1 + control RNA
SBF2-AS1 + miR-338-3p
SBF2-AS1 + miR-362-3p
SBF2-AS1 +miR-338-39/362-3p
N.S
Number of colonies
EV
SBF2-AS1
control RNA
miR-338-3p
miR-362-3p
B
EV
SBF2-AS1
SBF2-AS1 + control RNA
SBF2-AS1 + miR-338-3p
SBF2-AS1 + miR-362-3p
SBF2-AS1 + miR-338-39/362-3p
N.S
Percentage of positive cells (%)
C
A549
EV
SBF2-AS1
SBF2-AS1-miR-NC
SBF2-AS1+miR-338-3p
SBF2-AS1+miR-362-3p
SBF2-AS1+miR-338-3p+miR-362-3p
Relativeabsorbation (450nm)
Incubation time (hours)
D
H1299
EV
SBF2-AS1
SBF2-AS1+miR-NC
SBF2-AS1+miR-338-3p
SBF2-AS1+miR-362-3p
SBF2-AS1+miR-338-3p+miR362-3p
Relative absorbation 450(nm)
Incubation time(hours)

Figure S4. Venn plot of candidate target genes (A). ceRNA network driven by SBF2-AS1 (B).

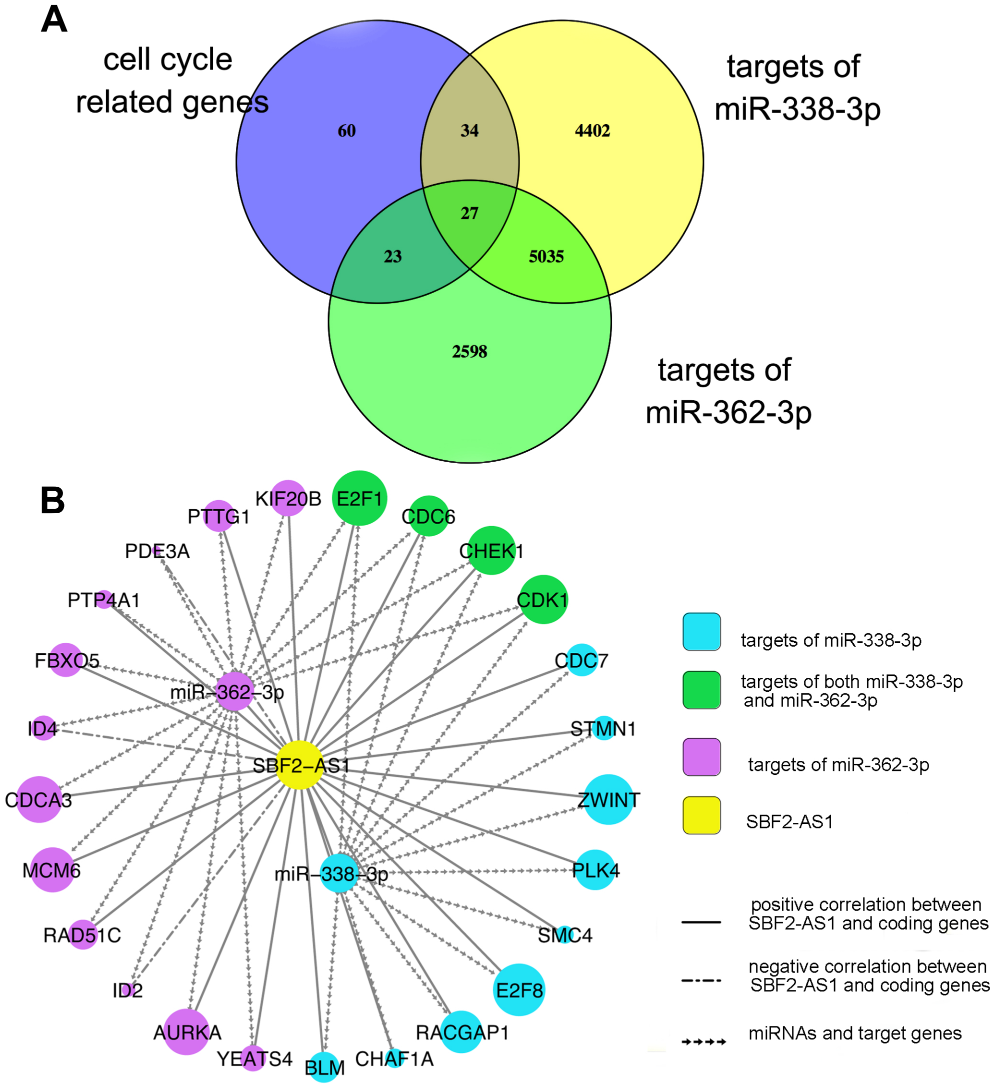

A
cell cycle related genes
targets of miR-338-3p
targets of miR-362-3p
60
34
4402
27
23
5035
2598
B
KIF20B
E2F1
PTTG1
CDC6
PDE3A
CHEK1
PTP4A1
CDK1
FBXO5
CDC7
miR-362-3p
ID4
STMN1
SBF2-AS1
CDCA3
ZWINT
MCM6
PLK4
miR-338-3p
RAD51C
SMC4
ID2
E2F8
AURKA
RACGAP1
YEATS4
BLM
CHAF1A
targets of miR-338-3p
targets of both miR-338-3p and miR-362-3p
targets of miR-362-3p
SBF2-AS1
positive correlation between SBF2-AS1 and coding genes
negative correlation between SBF2-AS1 and coding genes
miRNAs and target genes
